# Microfluidic Biosensors: Enabling Advanced Disease Detection

**DOI:** 10.3390/s25061936

**Published:** 2025-03-20

**Authors:** Siyue Wang, Xiaotian Guan, Shuqing Sun

**Affiliations:** Shenzhen International Graduate School, Tsinghua University, Shenzhen 518055, China; wang-sy23@mails.tsinghua.edu.cn (S.W.); guanxt22@mails.tsinghua.edu.cn (X.G.)

**Keywords:** microfluidics, biosensor, cancer liquid biopsy, pathogenic bacteria detection, POCT

## Abstract

Microfluidic biosensors integrate microfluidic and biosensing technologies to achieve the miniaturization, integration, and automation of disease diagnosis, and show great potential for application in the fields of cancer liquid biopsy, pathogenic bacteria detection, and POCT. This paper reviews the recent advances related to microfluidic biosensors in the field of laboratory medicine, focusing on their applications in the above three areas. In cancer liquid biopsy, microfluidic biosensors facilitate the isolation, enrichment, and detection of tumor markers such as CTCs, ctDNA, miRNA, exosomes, and so on, providing support for early diagnosis, precise treatment, and prognostic assessment. In terms of pathogenic bacteria detection, microfluidic biosensors can achieve the rapid, highly sensitive, and highly specific detection of a variety of pathogenic bacteria, helping disease prevention and control as well as public health safety. Pertaining to the realm of POCT, microfluidic biosensors bring the convenient detection of a variety of diseases, such as tumors, infectious diseases, and chronic diseases, to primary health care. Future microfluidic biosensor research will focus on enhancing detection throughput, lowering costs, innovating new recognition elements and signal transduction methods, integrating artificial intelligence, and broadening applications to include home health care, drug discovery, food safety, and so on.

## 1. Introduction

**Figure 1:**
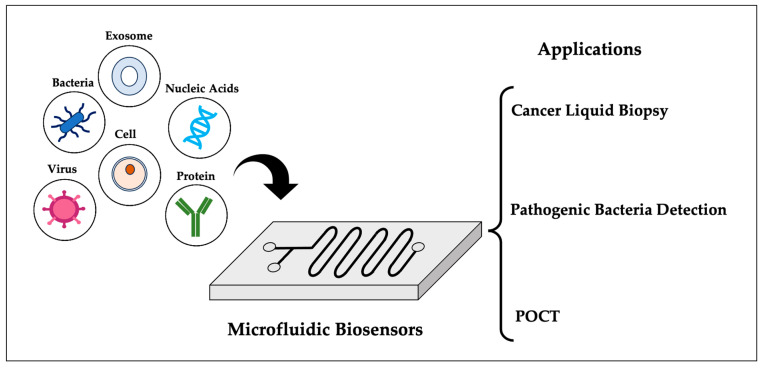
An overview of the application of microfluidic biosensors in disease detection.

Microfluidic biosensors involve the collection of sensing elements on microfluidic chips to achieve the integration of detection devices used in fluid infusion, control over signal reception, conversion, and presentation [1]. These devices integrate innovations from chemistry, biology, optics, electronics, and other disciplines to achieve the miniaturization, integration, and automation of sample handling and analysis processes. Microfluidic biosensors are characterized by the amalgamation of two fundamental technological components. The initial component is microfluidic technology, also termed “lab-on-a-chip”. The appellation “lab-on-a-chip” succinctly encapsulates the concept of miniaturizing chemical, biological, and medical laboratory processes onto a surface area generally spanning a few square centimeters [2]. The main feature of microfluidic chip is manipulating a small volume of fluid (10^−9^–10^−18^ L) to perform a specific function through a small dense network of flow channels (10^−6^–10^−9^ m) [3]. Microfluidic chips have several fundamental features compared to conventional macroscale experimental techniques: in microfluidic flow, inertial influence is negligible, viscous forces are the predominant factor, the Reynolds number is very small, and the fluid flows in a laminar manner within the pipelines. The specific surface area of the micro-pipeline is large, and the heat transfer in the system is rapid, so the reaction yield and conversion efficiency are improved compared with the macroscopic system under constant temperature conditions. The micro-pipeline scale is minute, reducing reagent consumption by 1–3 orders of magnitude compared to conventional methods, thereby significantly cutting reagent costs and waste liquid emissions [4]. Furthermore, the second key component of microfluidic biosensors is biosensing technology. This technology involves the detection of biological information through the conversion of biomolecules into signals that can be output and measured using acoustic, optical, electrical, and magnetic components, as well as other types [5]. The advantage of biosensors lies firstly in their high sensitivity compared with traditional large-scale analytical instruments. Biosensors target localized tiny areas where even weak biological signals can be captured and amplified. Secondly, biosensors often use label-free technology, which allows real-time detection over a long period of time, and the sensitive material is not consumed. As shown in Figure 1, the microfluidic biosensor organically combines the above microfluidic technology with biosensing technology, and on the same tiny chip, it not only realizes the sample preparation, separation, reaction, and cleaning by using microfluidic technology, but also collects, processes, and analyzes the signals through the biosensing technology, which realizes the whole detection process’s “from sample to result” function [6,7]. This review will focus on the applications of microfluidic biosensors in the fields of cancer liquid biopsy, pathogenic bacteria detection, and point-of-care testing (POCT), with a particular emphasis on their potential for enabling advanced disease detection.

## 2. Microfluidics and Microfluidic Biosensors

### 2.1. Materials for Microfluidic Chip Preparation

Microfluidics enables the precise control of fluids. By combining structures such as microchannels, microchambers, valves, and pumps, different microfluidic devices can be designed and fluids can be integrated into the microfluidic system and driven in an artificial way. In the microfluidic system, the surface properties of the chip’s own material can have a huge impact on the fluid due to the huge specific surface area at the microscale. In addition, complex microfluidic chips that meet the needs of practical applications can only be prepared if the physical indicators, such as the rigidity and elasticity of the material, meet the corresponding needs. Therefore, the selection of materials used for the preparation of microfluidic chips is crucial [8].

The advent of micromachining technology within the semiconductor industry precipitated the development of silicon/glass-based microfluidic chips, representing the first generation of such devices. Silicon-based microfluidic devices boast the advantage of corrosion resistance to organic solvents, coupled with superior thermal conductivity [9]. Conversely, glass-based microfluidic devices are prized for their high transparency, robust insulation properties, and minimal preparation costs [10]. Early silicon/glass-based chips found primary application in capillary electrophoresis. In addition, silicon/glass-based microfluidic chips have found application in biochemical reactions and the synthesis of materials. However, the preparation process requires the involvement of etching agents (e.g., hydrofluoric acid), which renders the preparation environment more hazardous. Furthermore, the coupling between silicon/glass materials must be carried out at high temperatures and high pressures, as well as in a clean environment, which further limits its wide application [11].

Thermoplastics, thermosets, and elastomers are the three distinct types of polymeric materials used to compose microfluidic chips, categorized based on their material properties [12]. Polydimethylsiloxane (PDMS) is the most widely used elastomeric material for the preparation of microfluidic chips at present. Its curing can be accomplished through temperature regulation, while its processing is enabled by soft lithography. The material’s capacity for facile expansion and contraction in response to external forces renders it particularly well suited for the integration of functional modules, such as pumps and valves, into microfluidic chips. PDMS has good permeability, which allows it to be used in cell culture, but also limits the accuracy of its quantitative detection in bioanalysis [13,14]. Thermosets such as SU-8 are stable when heated because the internal polymer molecules are crosslinked into a three-dimensional network structure. Most thermoset materials are thermally stable, solvent-resistant, and optically transparent. This is a clear advantage in the preparation of microfluidic materials. However, its high mechanical strength makes it difficult to process, limiting the development of thermoset materials for use in microfluidic chips. Thermoplastic materials such as polymethyl methacrylate (PMMA), polycarbonate (PC), polystyrene (PS), polyethylene terephthalate (PET), and polyvinyl chloride (PVC) are some of the common plastics used in life [15,16,17]. Industrially, they are softened by heating and shaped in molds, thus enabling low-cost mass production. For microfluidic chips, however, special designs are required depending on the material being prepared. Therefore, microfluidic chips based on thermoplastic materials are not common.

Hydrogel is a material that is produced by crosslinking hydrophilic polymers with an adjustable porous structure that allows for the diffusion of small molecules or biomolecules. Depending on the source, hydrogels are primarily classified as animal source, plant source, or synthetic. Among them, hydrogels of animal origin are notable for their high content of growth factors, which promote cell adhesion and proliferation, thus facilitating cell culture. However, the mechanical strength of hydrogels is inadequate, and they are susceptible to destruction during processing [18].

As for the paper-based microfluidic chip, it has an open structure and its channels are not closed. Cellulose paper is a porous material with a large number of groups on its surface for modification. Performing hydrophilic treatment induces the directional transport of fluids [19]. Paper chips do not require special coupling and are designed and processed by folding and embossing to form three-dimensional channels [20]. Paper chips do not require additional actuation devices, and the capillary force through the paper itself is sufficient. However, paper-based microfluidic chips are also subject to limitations, including low detection sensitivity and resolution. Furthermore, the process of droplet volatilization can result in inaccurate detection outcomes [21].

Capillary assembly devices are also common microfluidic chips, representing a new type of microfluidic device first proposed by Weitz et al. [22]. The capillary assembly technique used for microfluidic devices is notable for its ability to circumvent the need for intricate machinery or specialized experimental settings, as it can be manually executed. This modular approach to assembly confers a high level of operational versatility, enabling the manipulation of the geometry of the capillary assembly to effectively govern the fluid dynamics [23]. A salient benefit of this technology is the creation of three-dimensional (3D) axisymmetric flow paths that prevent fluid interactions with the channel walls. Conversely, conventional two-dimensional (2D) or quasi-2D channels, produced via etching or soft stencil replication, are prone to fluid–wall contact, which significantly influences fluid behavior. In order to address this issue in traditional methods, it is necessary to selectively modify the channel’s surface wettability, a process that is both labor-intensive and prone to causing wear. The capillary assembly method has been shown to streamline the production of microfluidic devices [24]. Nevertheless, due to its reliance on manual assembly, this method is less efficient in terms of production speed, making it more appropriate for academic research settings rather than for extensive industrial manufacturing. Table 1 summarizes the previously mentioned materials for microfluidic chip fabrication, comparing their respective strengths and weaknesses.

### 2.2. Surface Ttreatment of Microfluidic Chip Channels

Microfluidic chip-based biosensors usually require the surface treatment of the microfluidic flow channel, which is a key step in biosensor design and is important in several ways. Firstly, it can effectively prevent the non-specific adsorption of biological molecules in biological samples, such as proteins and DNA, which can lead to signal interference and affect the accuracy and reliability of detection results. Secondly, the biocompatibility of the microfluidic chip can be improved by surface treatment to avoid the interaction between the chip material and the biological sample, which can lead to the denaturation or loss of activity of the sample. In addition, surface treatment can also enhance signal intensity, such as immobilizing specific biorecognition elements (e.g., antibodies, nucleic acid aptamers, etc.) on the surface of the channel, to achieve the specific capture and enrichment of target molecules, thus improving detection sensitivity. Finally, through different surface treatments, the multifunctionality of microfluidic chip channel surfaces can be achieved, such as the simultaneous detection of multiple biomolecules, cell culture, drug screening, etc.

#### 2.2.1. Anti-Pollution Protection Treatments

To combat biological contamination in microfluidic systems, anti-pollution treatments are essential [25,26,27]. Trace biomolecule adsorption can impair device performance or block channels. Common treatments involve coating with inert proteins like BSA [28] or polymers such as PLL-g-PEG [29], but these methods can lead to inhomogeneous coatings and clogging in nanochannels. Fukuda et al. introduced the silylation of monomers, specifically choline phosphate groups, to passivate silica nanochannels, reducing blockage risks [30]. Lipid bilayers formed by lipid vesicle rupture offer long-term protection but may not suffice against biofluids [31]. Andersson et al. developed a method for the covalent grafting of PEG polymers using aminopropyl siloxane click chemistry, providing a more homogeneous coating with ultra-low contamination levels in crude serum [26].

#### 2.2.2. Functionalization for Bio-Selectivity

Nanochannel surfaces can be modified with biorecognition elements for selective binding, enhancing functionality. Shirai et al. used photolithography to pattern silane groups for antibody immobilization and coated nanochannels with PEG to prevent non-specific adsorption [32,33]. This technique, using UV light and a chromium glass mask, allows for mild-temperature bonding while preserving APTES stability. Despite PEG’s minimal impact on antibody density, the balance between contamination prevention and functionalization is crucial [34]. Andersson et al. grafted biotinylated PEG on silica as a versatile approach to linking biotinylated bioreceptors via streptavidin, paving the way for the use of multifunctional coatings in clinical sample analysis [26].

### 2.3. Principles of Microfluidic Biosensor Technology

The technical principle of a microfluidic biosensor involves three aspects, namely, a biosignal-sensitive element affinity mode, a sensor signal conversion mode, and microfluidic control technology. The latter of these covers multidisciplinary knowledge of biochemistry, electromechanical processing, fluid dynamics, micro/nano-processing and materials chemistry, amongst other subjects. In the following discussion, the technical principle of microfluidic biosensor will be elaborated from the perspective of microfluidic chips.

#### 2.3.1. Flow Channel Microfluidic Biosensors

**Figure 2:**
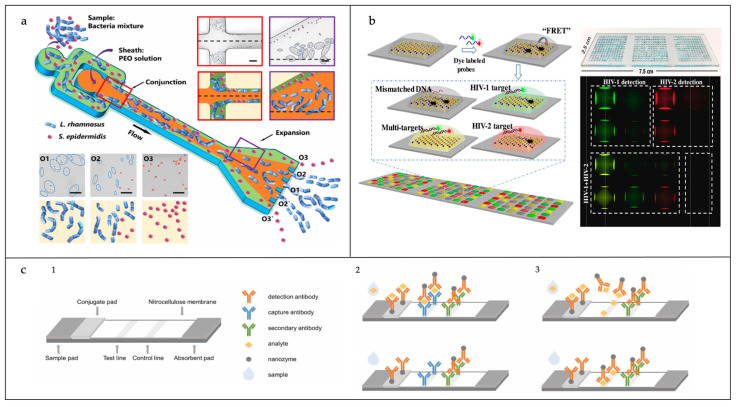
Microfluidic biosensors as classified according to principles of microfluidic technology. (**a**) Schematic of continuous flow microfluidic biosensors. Reproduced with permission from Ref. [35]. Copyright 2023 Sensors and Actuators B: Chemical. (**b**) Schematic of digital microfluidic biosensors. Reproduced with permission from Ref. [38]. Copyright 2020 ACS Applied Materials & Interfaces. (**c**) Schematic of paper-based microfluidic biosensors. Reproduced with permission from Ref. [39]. Copyright 2024 Nanotoday.

Flow channel microfluidic biosensors are the most used type at present, and can be further divided into the continuous flow type and the droplet type. Continuous flow microfluidic pipelines are closed pipelines, and the flow and cut-off of the liquid is controlled by syringe pumps and air valves, thus realizing continuous flow and precise control of the liquid. Continuous flow microfluidic chips can now accurately handle nanogram quantities of liquid, thus enabling research at the single-cell or even single-molecule level [35,36]. As shown in Figure 2a, Zhang et al. developed a continuous flow microfluidic biosensor based on a viscoelastic fluid, which enabled the size-dependent separation of *Lactobacillus* from *Staphylococcus epidermidis* or *Escherichia coli* by adjusting the physical parameters of the fluid [35]. Droplet microfluidic chips, through the droplet generator, can produce droplets with high throughput. The nature of the droplets is that they are encapsulated in the oil phase or other materials, with the water phase forming the droplets. Changing the contents of the water phase allows each droplet to be regarded as an independent microreactor. Due to the small size of these individual droplets, the amount of sample required is minimal, making them suitable for high-throughput screening and sensitive bioassays. Additionally, since the encapsulated samples within the droplets are stable, evaporation and mutual contamination phenomena can be effectively controlled. This stability has led to their widespread use in applications such as digital polymerase chain reaction (PCR) and other fields. For example, Li et al. used a droplet-separated single-cell microfluidic biosensor to efficiently separate and localize individual cells, enabling the manipulation and analysis of individual cells and providing a more comprehensive assessment of nanomaterials for use in cancer therapy [37]. The flow channel microfluidic chip is suitable for a wide range of biosensor signal conversion types due to its design flexibility and expandability, including optical-based surface-enhanced Raman spectroscopy, acoustic-based quartz crystal microbalance, acoustic surface waves, and electrochemical impedance based on electrochemistry.

#### 2.3.2. Digital Microfluidic Biosensors

Digital microfluidics refers to the implementation of droplet driving within the microfluidic chip through electrical, acoustic, thermal, and chemical methods to reduce the error caused by human operation [40], and the common methods are dielectric electrophoresis, dielectric wetting, thermal capillary effect, acoustic surface wave, electrostatic force method, etc. [41]. Its dielectric wetting method has low driving energy and simpler peripherals compared to other methods and is most widely used in the digital microfluidic chip manipulation of droplets. Digital microfluidics manipulates independent droplets on a dielectric-coated electrode array by applying a series of potentials to the electrode array, and switching and adjusting the electrodes through digital encoding, thus realizing the driving, merging, mixing, and separating of the droplets. Digital microfluidics can work on one or two control panels and have the ability to handle droplets ranging in size from the picoscale to the microscale, resulting in reduced reagent consumption and more automated reactions. Unlike flow channel microfluidics, digital microfluidics does not place high demands on pump valves and mechanical mixing devices and can control the driving of individual droplets. In addition, the digital microfluidic control of droplets can be changed through programming to meet different experimental requirements and customized according to the program to improve prospects for application and enable a wider range of applications. Another major advantage of digital microfluidics is the reduction in clogging in liquids containing insoluble substances through electrode actuation. Since digital microfluidics integrates electronic components, it can be combined with most biosensing technologies, making digital microfluidic biosensors powerful microfluidic tools for controlling complex biological assays, as shown in Figure 2b [38,42].

#### 2.3.3. Paper-Based Microfluidic Biosensors

Paper-based microfluidic chip pipelines are open as opposed to the closed flow channels of microfluidic chips. Paper is a porous matrix made of cellulose, which has good liquid absorption capacity. When hydrophobized, the liquid will flow precisely in the hydrophilic ducts by capillary action. Paper-based microfluidic chips have a bright future in the field of portable, low-cost, and immediate detection (Figure 2c) [39,43]. Immunochromatographic test strips, commonly utilized in clinical diagnostics, operate on a similar principle. However, these strips lack the precision for accurate quantification and do not support multiplex analysis. Paper-based microfluidic chips solve this problem very well. The preparation of paper-based microfluidic chips is very simple and, in general, the use of paper-based microfluidic chips is feasible as long as they can form hydrophobic patterns. Currently, there are two main types of paper-based microfluidic preparation methods: the flatbed printing method has a high resolution but is more expensive; the method of directly printing the hydrophobic barrier channel is less demanding relative to instrumentation [44]. The 3D microfluidic paper analysis system (3D μPAD) developed by Martinez et al. is made of layers of paper, stacked in a specific pattern, as well as water-resistant double-sided adhesive, which connects stacked pipes to enable the vertical flow of liquid into the microfluidic chip through the double-sided adhesive [19]. By connecting the stacked tubes with double-sided adhesive, we ensure the liquid flows vertically through the tubes of different layers. The blood glucose and protein composition of four people can be measured simultaneously, and the whole process only takes 2 min. The paper-based microfluidic chip is inexpensive, easy to operate, and does not require an external force to drive it, but at the same time, the limitations of the paper-based microfluidic chips have limited its application. For example, because the driving force relies on the laminar action to actually reach the test area of only 50% of the sample volume, the open tubes of the paper chip can be easily heated when heating. For example, because the driving force is dependent on chromatography, only 50% of the sample volume is actually reached. The open tubing of paper-based microfluidic chips is susceptible to the evaporation of the solution when heated, and is more vulnerable to environmental factors.

## 3. Applications of Microfluidic Biosensors

### 3.1. Cancer Liquid Biopsy

In recent years, liquid biopsy technology has sparked a profound change in the field of clinical oncology due to its revolutionary potential. As an innovative non-invasive test, liquid biopsy technology opens up a new era of cancer diagnosis by finely analyzing samples of body fluids such as blood, saliva, and urine. The core of the technology lies in the precise identification of biomarkers that are closely associated with cancer, including circulating tumor cells (CTCs), circulating tumor DNA (ctDNA) fragments, microRNA (miRNA), and exosomes released by tumor cells, among others. The application of microfluidic biosensing devices in the field of liquid biopsy has greatly enhanced the utility and accuracy of this technology. Microfluidics, with its miniaturization, integration, and automation, provides a powerful tool for liquid biopsy; in particular, its ability to efficiently process and analyze minute quantities of biological samples makes it possible to detect low-abundance biomarkers. Droplet-based microfluidic biosensors achieve the rapid separation, enrichment, and detection of cancer-related molecules in body fluid samples through the precise control of microfluidics, which greatly improves the sensitivity and specificity of liquid biopsies and provides powerful technical support for the early detection, precise treatment, and prognostic assessment of cancer. A brief summary of this next section is shown in Table 2.

#### 3.1.1. CTCs

The term “tumor heterogeneity” refers to differences in the phenotypes or genes of CTCs that occur during tumor development. This phenomenon is not only observed between different tumors, but may also occur between cells within the same tumor tissue, which poses a significant challenge to tumor therapy. The conventional analytical tools employed in such contexts have a tendency to infer cell status by analyzing the average level of the cell population. This approach results in a considerable loss of information pertaining to individual cells. Consequently, there exists a need for sophisticated analytical tools that are able to detect and measure the unique characteristics of individual cells. Such single-cell high-throughput analysis techniques are able to reveal the heterogeneity of cell populations, to identify rare cell subpopulations, and to explore the unique characteristics of individual cells. Conventional single-cell isolation techniques, encompassing laser capture microdissection (LCM) [45], fluorescence-activated cell sorting (FACS) [46], and micromanipulation [47], are plagued by several inherent limitations. These include bias towards specific samples, the requirement for high sample concentrations, constrained throughput, and the potential for cumbersome manipulation. Moreover, these techniques are vulnerable to contamination and exhibit low amplification efficiency. This has led to an urgent need for the development of new single-cell separation techniques that are both accurate and devoid of contamination. Such new techniques must also be capable of handling large quantities of samples with ease, thus facilitating more efficient bioanalysis. The introduction of droplet microfluidics has opened a new chapter in liquid biopsy research by greatly simplifying the separation of single cells. This technique is known for its simplicity, automation, and high throughput in droplet generation. The ability of single cells to be precisely encapsulated into tiny droplets ensures the independence and efficiency of the assay while avoiding external interference. These droplets offer a highly controlled and contamination-free microenvironment, facilitating the rapid concentration of the contents, which is essential for efficient and sensitive liquid biopsy analysis. The high rate of droplet generation and the concentration of contents provide researchers with powerful tools for large-scale, high-precision single-cell analysis. Single cells, encapsulated in droplets, provide the basis for further complex analyses. These analyses include, but are not limited to, cellular imaging, immunoassays, PCR technology assays, and next-generation sequencing (NGS) technologies.

**Figure 3:**
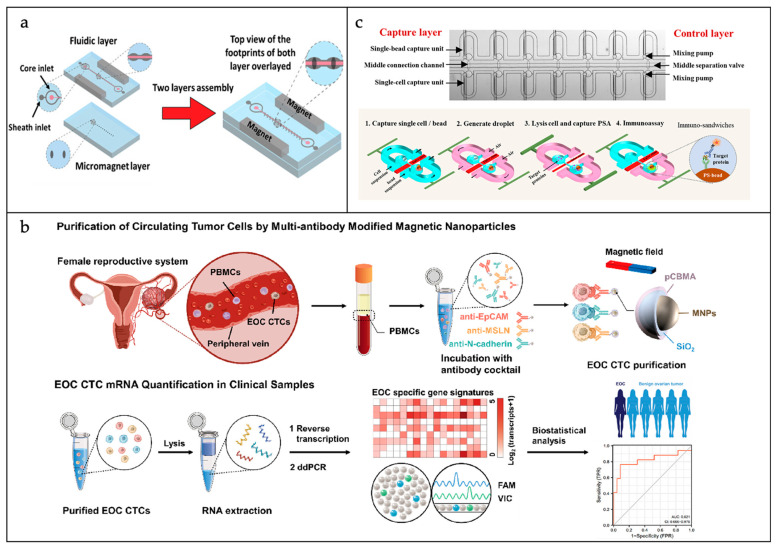
Microfluidic biosensors used for CTCs detection. (**a**) A schematic of the bilayer microfluidic chip. Reproduced with permission from Ref. [48]. Copyright 2023 Small. (**b**) A schematic of EOC CTCs RNA purification and quantification. Reproduced with permission from Ref. [50]. Copyright 2023 ACS sensors. (**c**) The working principle of the droplet-based microfluidic platform. Reproduced with permission from Ref. [51]. Copyright 2022 Langmuir.

To date, the application of microfluidics in the field of CTC separation has been studied widely. For example, Seyfoori et al. developed a bilayer microfluidic platform that combines a smart antibody-coupled magnetic nano/hybrid microgel and a magnetic microfluidic chip [48]. As shown in Figure 3a, the bottom of the chip contains an array of miniature magnets for generating a local magnetic field to guide magnetically labeled cells into the capture microchamber. The smart magnetic nano/hybrid microgel incorporates antibodies that specifically bind to tumor cells and release fluorescent dyes through temperature-responsive behavior, enabling in situ staining and the real-time monitoring of tumor cells. They optimized the chip design and capture parameters using finite element simulation to improve capture efficiency and purity. The results show that the platform achieves higher capture efficiency and purity than conventional magnetic separation methods and can easily release captured cells for downstream analysis. In addition, the smart magnetic nano/hybrid microgel enables in situ staining and the real-time monitoring of the captured cells without additional manipulation steps. Akbarnataj et al. developed a novel microfluidic device based on size-dependent cell sorting for the isolation of CTCs from peripheral blood samples [49]. The initial design of the microfluidic device incorporated a trapezoidal cross-section and an elliptical helix configuration, meticulously engineered to facilitate the separation of CTCs from white blood cells (WBCs) through the application of inertial and Dean drag forces. In the assessment of the separation potential and quality of the devices, we conducted a series of numerical simulations. Fabrication was then undertaken to investigate the effects of varying the curvature and trapezoidal cross-section on the separation of CTCs at different flow rates (0.5 mL/min to 3.5 mL/min). The findings indicated that a flow rate of 2.5 mL/min yielded the optimal separation efficiency in the proposed device, a result that aligns with the numerical analysis. In this study, the purity values of CTCs ranged from 88% to 90%, which demonstrated the high ability of the proposed device to separate and enrich CTCs. In addition, the device exhibited 90% separation efficiency in isolating MCF-7 cells and was able to maintain sufficient cell viability.

The analysis of CTCs using genomic methods is recognized as a pivotal component of liquid biopsy protocols. The capacity for genetic variations, encompassing deletions, mutations and rearrangements, to influence the uncontrolled proliferation, infiltration, and metastasis of tumor cells makes this approach imperative. However, the presence of genetic mutations can be obscured by a substantial non-mutated background, thereby hindering their detection. In the realm of microfluidic sensing device-based liquid biopsies, the initial step entails the isolation of CTCs, followed by the extraction of their genome through a process of cell lysis. This extracted genome is then subjected to rigorous analysis using a variety of techniques, including PCR, mass spectrometry (MS), and nucleic acid sequencing. To facilitate genomic analysis at the single-cell resolution, Ruan et al. engineered an advanced, integrated digital microfluidics (DMF) platform tailored for single-cell whole-genome sequencing (WGS) [52]. The platform is equipped with a butterfly trap, which functions to physically separate individual cells into nanoliter-sized droplets. An EWOD driver is incorporated into the system to facilitate control of the droplets during the process of cellular membrane disruption, comprehensive genomic amplification, and the following sequencing steps. It has been demonstrated that this platform is capable of detecting copy number variants (CNVs), as well as single-nucleotide variants (SNVs) at a minimal partition size of 150 kb, accompanied by an allelic loss (ADO) rate of 5.2%. However, the platform’s sample analysis throughput is limited due to the number of structures captured in a single cell. In order to overcome this limitation, Ruan et al. further devised a DMF-based approach for single-cell MS analysis, enabling the comprehensive mutation profiling of solitary CTCs [53]. The method combines a wettability-based capture process with the hydrodynamic tuning of cell distribution by encapsulating individual CTCs within droplets on a DMF device. Cell lysis and whole-genome amplification are then performed. Following primer amplification, MALDI-TOF MS is utilized for the simultaneous detection of multiple mutations in individual CTCs, leveraging the inherent quality disparities among diverse DNA sequences. The DMF-scMS platform has demonstrated its efficacy in detecting KRAS genetic alterations within a diverse array of CTCs sampled from a group of five individuals with colorectal carcinoma, yielding a perfect rate of detection.

In clinical practice, the assessment of RNA levels in CTCs has important prognostic value [54]. At present, RNA detection techniques comprise reverse transcription PCR (RT-PCR) and RNA sequencing. Amongst these, RT-PCR is regarded as the gold standard, a technique the incorporates a reverse transcription step in addition to the conventional PCR. Ma et al. developed a novel purification method for CTCs, as shown in Figure 3b [50]. This methodology is based on multiple antibody-modified magnetic nanoparticles, utilizing molecular analysis for the precise and accurate detection and monitoring of epithelial ovarian cancer (EOC). They used MNPs to isolate EOC CTCs from whole-blood samples from clinical patients and used droplet digital PCR (ddPCR) to quantitatively purify nine EOC-specific mRNAs from the CTCs. Using multivariate logistic regression modeling, they generated EOC CTC scores for each sample based on the transcripts of the nine genes. The results demonstrated that the assay exhibited excellent diagnostic performance (AUC = 0.96) in terms of differentiating EOC from benign ovarian tumors. Furthermore, dynamic fluctuations in EOC CTC scores were observed in treated patients, thereby underscoring the assay’s capacity for EOC monitoring. In addition to ddPCR, the droplet-based approach can be used alongside RNA-seq techniques. Xu et al. introduced a DMF system for single-cell mRNA sequencing that offers an economical method for mRNA analysis [55]. This system leverages the principles of hydrophilic and hydrophobic interactions to facilitate the generation of sub-droplets and to ensure accurate reagent blending. The technique, offering low reagent use and high sensitivity, shows potential in gene transcript and cell type analysis, but requires electrode number enhancement for higher throughput.

Single-cell proteomics is as important as genomics and transcriptomics. The principal challenges associated with single-cell proteome analysis pertain to the scarcity of CTCs, low protein levels, and significant background noise. DNA or RNA can be directly amplified to improve signal clarity; however, this approach is not viable for proteins. Consequently, the employment of droplet-based high-throughput microfluidics, which can enclose single cells in ultra-small droplets, has emerged as a promising solution to address the limitations of conventional techniques such as flow cytometry when analyzing single-cell secreted proteins. This approach has the potential to enhance the performance of single-cell protein assays by overcoming the major challenges associated with traditional methods and thereby improving the signal-to-noise ratio. Khajvand et al. developed an advanced droplet microfluidic platform featuring antibody barcode spatial arraying, designed for the simultaneous detection of single-cell cytokine release [56]. The platform efficiently sequesters cells into droplets with over 80% capture efficiency and detects four cytokines, including IL-8, MCP-1, MIP-1b, and TNF-α/IL-10, following four-hour incubation with high sensitivity via the sandwich enzyme-linked immunosorbent assay (ELISA). The technique is applied to analyze human tumor cell lines and clinical samples, assessing protein secretion and heterogeneity, with matrix metalloproteinases (MMPs) highlighted as potential biomarkers for cancer metastasis detection. In an alternative study, as shown in Figure 3c, Liu et al. constructed a droplet-based microfluidic system to perform the quantitative profiling of proteins within the cellular interior at the single-cell level [51]. The capture efficiency for individual CTCs and detector beads in the two chambers following disconnection was as high as 75%. The generation of twin droplets (160 pL in volume), each encapsulating a solitary cell and a lone assay bead, was achieved through the application of retrograde airflow. Subsequent to this, the single cells are lysed. Upon the subsequent connection of the two chambers, the liberated prostate-specific antigen (PSA) is identified by the sensing beads. Their assay’s sensitivity is demonstrated by its capacity to detect single-cell prostate-specific antigen using a sandwich immunoassay with a limit of detection (LOD) of 0.3 nM, as measured by the change in fluorescence signal.

#### 3.1.2. ctDNA

ctDNA is defined as a tumor-derived free DNA fragment present in body fluids, with particular prevalence in plasma. Within the realm of liquid biopsy, ctDNA has arisen as a hopeful indicator, providing an extensive overview of cancer-related genetic modifications, such as single-nucleotide variants, epigenetic modifications, copy number alterations, and methylation signatures, among additional changes. Furthermore, ctDNA is amenable to extraction and storage in comparison to other tumor markers.

**Figure 4:**
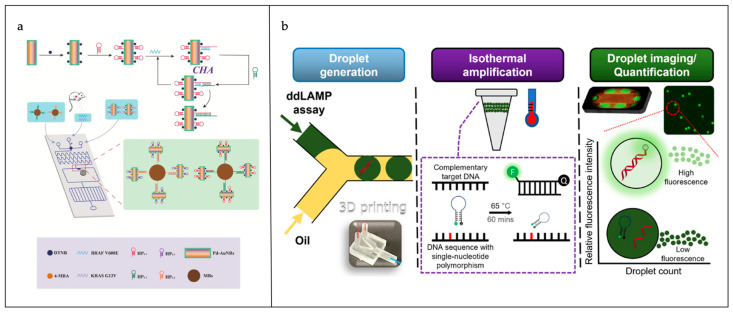
Microfluidic biosensors for ctDNA detection. (**a**) A schematic of a dual-signal amplification strategy based on the pump-free SERS microfluidic chip. Reproduced with permission from Ref. [59]. Copyright 2022 Biosensors and Bioelectronics. (**b**) Techniques for executing ddLAMP with an assay that utilizes an MB as a sequence-specific probe for the identification of SNPs. Reproduced with permission from Ref. [61]. Copyright 2022 Analytical Chemistry.

In the field of liquid biopsy, ddPCR stands out as the premier technique for detecting ctDNA, using thousands of droplets to dilute and isolate single cells or molecules. Subsequently, the template is subjected to PCR amplification, followed by the analysis of the droplet’s fluorescence signal using a droplet analyzer [57]. The integration of microfluidic sensing devices with ddPCR has been demonstrated to enhance the sensitivity of the latter technique. Ou et al. developed an integrated droplet digital detection (ID3D) setup that integrates three distinct technologies: droplet partitioning, fluorescence-multiplexed PCR, and three-dimensional droplet counting technology [58]. The primary function of this system is the detection of ultra-rare ctDNAs from an extensive range of biological samples. In the IC3D system, picolitre-sized droplets encapsulating the target molecules are produced using the flow-focusing technique and subsequently amplified off-chip through thermal cycling. These droplets are subsequently gathered en masse for efficient, high-throughput three-dimensional particle examination. The IC3D ddPCR system is distinguished by its capacity to detect oncogenic KRAS G12D mutant alleles, with a detection sensitivity between 0.00125% and 0.005%, while maintaining a zero percent false-positive rate. Furthermore, the IC3D system is capable of analyzing a 1 mL blood sample within minutes, whereas the Bio-Rad ddPCR system requires a duration of 2 h. As shown in Figure 4a, Cao et al. devised a twin-signal enhancement approach, utilizing a pumpless surface-enhanced Raman spectroscopy (SERS) microfluidic device in conjunction with a catalytic hairpin assembly (CHA) methodology for the swift and highly sensitive identification of ctDNA associated with non-small-cell lung carcinoma. They utilized Pd-Au nanorods as SERS probes and CHA as a method to amplify ctDNA [59]. This strategy uses Pd-Au nanorods as SERS probes, and the CHA reaction binds the probes to the target ctDNA and aggregates them in the detection area of the chip to achieve double amplification of SERS signals. The detection limit in the serum can reach the aM level. Further, Cao et al. developed an SERS assay based on a pump-free high-throughput microfluidic chip and a cascade signal amplification strategy for the detection of gastric cancer-associated ctDNA [60]. The chip combines Cu_2_O octahedral and Au nanobowl arrays as SERS active substrates and utilizes CHA and hybridization chain reaction (HCR) strategies for signal amplification. The detection limits of the chip reached 1.26 aM and 2.04 aM for PIK3CA E542K and TP53, respectively, and the whole detection process could be completed within 13 min. In addition, the microarray technology was effectively utilized for the identification of ctDNA in a hormonal murine model, exhibiting strong concordance with the findings of quantitative reverse transcription polymerase chain reaction (qRT-PCR), thereby illustrating its potential utility in the preliminary diagnosis and treatment monitoring of gastric adenocarcinoma.

The employment of thermal amplification techniques has demonstrated the ability to streamline the thermal cycling procedure, and these methods are set to gain prominence as substitutes for PCR in the field of circulating tumor DNA (ctDNA) identification [62]. The fusion of microfluidic detection systems with isothermal amplification methods for the identification of ctDNA has been reported. As shown in Figure 4b, Hsieh et al. created a diagnostic assay based on digital loop-mediated isothermal amplification (ddLAMP) that unites the principles of molecular beacon (MB) detection, the application of additive manufacturing for the creation of microdroplets, and the utilization of mobile phone-based imaging technology, thereby enhancing the ability to identify specific DNA sequences [61]. The ddLAMP assay is executed within droplets produced by a three-dimensional printed microfluidic device with a Y-shaped junction configuration. This innovative approach leverages a 3D printer with a Y-jointed apparatus for droplet creation. The LAMP reaction, integral to this methodology, is conducted in the presence of MBs, which act as sequence-specific fluorescent indicators, enabling accurate detection and quantification. The enhancement in fluorescence intensity, indicative of the assay’s performance, is quantitatively assessed using a fluorescence microscope fitted with an EGFP-specific filter, paired with a smartphone camera setup. The MB-ddLAMP technique has been verified for the detection of single-nucleotide variations (SNVs), achieving a sensitivity of 1% and a detection threshold of 4.39 copies/μL using the OmpW DNA target.

#### 3.1.3. miRNA

MiRNA, belonging to the category of intrinsic non-protein-coding RNAs, fulfills crucial biological roles through the modulation of gene expression during transcription, RNA maturation, and protein synthesis. Numerous studies have shown that abnormal miRNA expression levels are closely related to tumor development [63]. Identifying miRNA, specifically expressed in different types of cancer, is important for clinical diagnosis and treatment.

**Figure 5:**
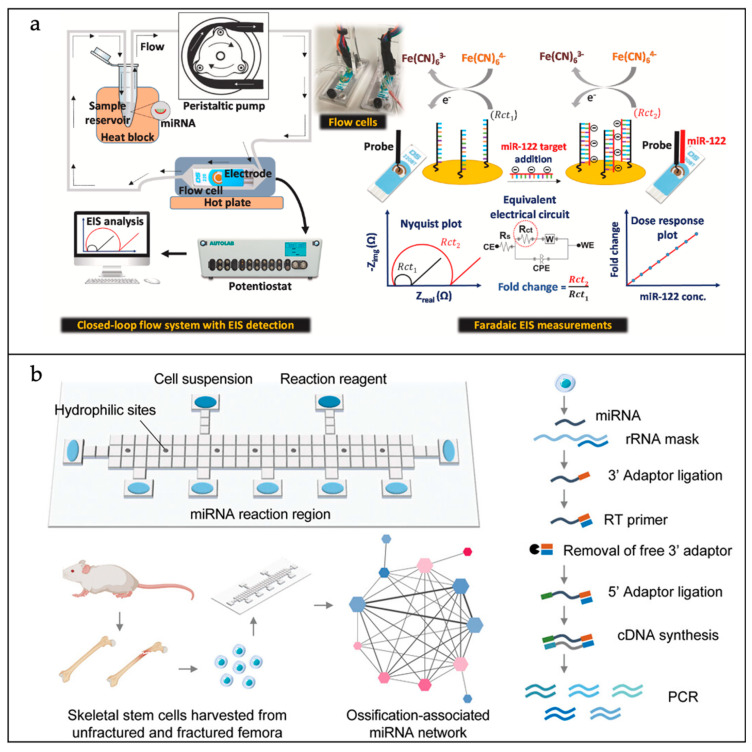
Microfluidic biosensors for miRNA detection. (**a**) Electrochemical impedance spectroscopy (EIS) closed-loop flow system for direct miRNA detection. Reproduced with permission from Ref. [65]. Copyright 2023 Biosensors and Bioelectronics. (**b**) Schematic of Hiper-seq for highly multiplexed single cell miRNA sequencing. Reproduced with permission from Ref. [66]. Copyright 2024 Small Methods.

An enzyme-free digital droplet autocatalytic hairpin assembly (ddaCHA) system was developed by Chen et al. for the quantification of miRNA at a single-molecule resolution [64]. A notable feature of the ddaCHA system is its capacity for amplification at room temperature, obviating the necessity for heating. The system’s sensitivity is demonstrated by its ability to detect changes in fluorescence, induced by hybridization over a linear range from 1 pM to 10 nM, with an LOD of 0.34 pM. High-throughput tracking calculations of fluorescent droplets, performed using an in-house-developed script, reduced the LOD to 10 fM. As shown in Figure 5a, Roychoudhury et al. developed an electrochemical impedance spectroscopy (EIS)-based microfluidic biosensing device for the detection of circulating miR-122. This was used for the diagnosis of drug-induced liver injury (DILI) [65]. Sequence-specific peptide nucleic acid (PNA) probes were utilized to modify screen-printed gold electrodes, and the successful immobilization of the PNA probes was confirmed through the characterization of the electrode surfaces by atomic force microscopy (AFM), scanning electron microscopy (SEM), energy-dispersive X-ray spectroscopy (EDX) and cyclic voltammetry (CV). Concurrently, a closed-loop microfluidic system was designed and characterized with the objective of enhancing hybridization efficiency and reducing sample volume requirements. The biosensor exhibited high specificity for miR-122, with an LOD of 50 pM.

Moreover, the examination of miRNA within individual cells with single-cell precision is essential for deciphering the diversity among cells. Chen et al. developed a single-cell analysis technique based on a microfluidic chip platform, enabling the concurrent measurement of the precise copy numbers of various types of miRNA present within an individual cell [67]. This innovation integrates DNA hairpin probe technology, nucleic acid signal enhancement, and microchip-based electrophoretic separation (MCE) to accomplish the highly delicate and selective detection of miRNA within cellular samples. Using miRNA-21 and miRNA-141 as models, they verified the feasibility of the method and successfully applied it to the analysis of cell lysates from different cell types, achieving the accurate determination of the absolute copy number of miRNA-21 and miRNA-141 in individual cells. In addition, the single-cell sequencing of miRNA using microfluidic sensing devices has also been reported. Chen et al. developed a novel microfluidic platform called Hiper-seq, which aims to address the limitations of traditional single-cell miRNA sequencing techniques (Figure 5b) [66]. Hiper-seq is based on digital microfluidics (DMF) technology, and can isolate and process multiple single cells simultaneously by designing hydrophilic microstructures and flexibly manipulating addressable droplets. The nanoliter reaction volume on the DMF chip improves miRNA detection and reduces the cost of reagents, and the pre-programmed motorized control automates the operation process, reduces the number of manual operations, and improves the reproducibility. Hiper-seq has effectively delineated the heterogeneity in miRNA expression profiles across various cell types. Furthermore, it has successfully identified miRNA implicated in bone repair and regeneration processes, thereby offering an innovative approach for single-cell miRNA sequencing studies.

#### 3.1.4. Exosome

Exosomes, measuring between 30 and 200 nanometers in diameter, are durable, membrane-bound vesicles emitted by cancerous cells into the extracellular environment. They are detectable in the bloodstream and function as transporters of various biological materials originating from the tumor, such as genetic material (DNA, RNA) and proteins. As essential mediators of cellular communication, these extracellular exosomes are massively produced and shed, playing a significant role in the progression of cancer by influencing processes like tumor expansion, infiltration, and the development of metastatic lesions. Unlike CTCs and ctDNA, circulating exosomes are found in copious amounts in a variety of bodily fluids such as urine, saliva, plasma, and serum. These particles exhibit significantly elevated expression in patients with early-stage cancer, surpassing the levels observed in CTCs and tumor antigens. This points to their potential as a highly sensitive indicator of tumor progression. Additionally, exosomes have the ability to encapsulate nucleic acids within their vesicular structure, which addresses the issue of rapid degradation faced by ctDNA and RNA in the bloodstream. As a result, circulating exosomes are regarded as promising diagnostic, prognostic, and monitoring biomarkers for early-stage cancer due to their prevalence and stability, as well as the ease with which they can be isolated from bodily fluids [68].

**Figure 6:**
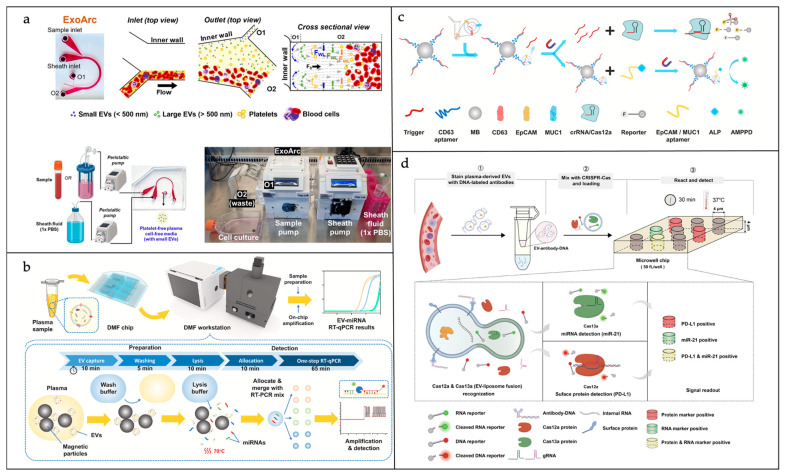
Microfluidic biosensors used for exosome detection. (**a**) ExoArc technology for PFP extraction from blood to isolate EVs. Reproduced with permission from Ref. [69]. Copyright 2024 ACS Nano. (**b**) Outline and operational sequence of suggested compact DMF system used to detect EV miRNA. Reproduced with permission from Ref. [70]. Copyright 2025 Biosensors and Bioelectronics. (**c**) Diagram of CRISPR/Cas12a and aptamer–chemiluminescence assay (CACBA) used to measure exosomes with cancer-related proteins. Reproduced with permission from Ref. [71]. Copyright 2024 Biosensors and Bioelectronics. (**d**) Schematic of ddSEE System. Reproduced with permission from Ref. [72]. Copyright 2024 ACS Nano.

Microfluidic chip-based exosome separation technology is maturing. As shown in Figure 6a, Leong et al. developed a novel microfluidic technology called ExoArc for the rapid and efficient separation of platelet-free plasma (PFP) from blood for RNA and extracellular vesicle (EV) analysis [69]. ExoArc has the advantages of high-purity PFP separation, high throughput, simple operation, and reduced platelet contamination and can be used in combination with Size Exclusion Chromatography (SEC) to rapidly separate EVs and reduce protein contamination. ExoArc can also be used to isolate EVs directly from low-volume cell culture media and for the real-time monitoring of EV secretion.

The deployment of ddPCR as a downstream analysis tool has become a prevalent practice in the field of exosomal nucleic acid detection. For instance, Cui et al. engineered a microfluidic apparatus that merges flow focusing with co-fluidization techniques, facilitating the one-step RT-PCR identification of miRNA within singular exosome specimens [73]. Their study meticulously isolated exosome fractions through high-speed centrifugal separation. Following this, they tagged the purified exosome samples with CD63-targeted MBs. Utilizing a novel co-fluidization technique, they encapsulated the necessary components for RT-PCR, including the reaction mix and lysis reagents, within individual droplets. The assay’s detection threshold, as highlighted by Cui’s findings, is remarkably sensitive, capable of identifying a single molecule of synthetic has-miR-21-5p within each droplet. In the context of clinical samples from individuals with lung cancer, the presence of exosomal RNA within positive droplets was confirmed after 30 cycles of PCR amplification. Tong et al. developed a platform that uses digital microfluidics (DMF) to manipulate tiny droplets for isolation, lysis, template distribution, and the real-time PCR detection of EVs (Figure 6b) [70]. They improved the sensitivity and specificity of the assay and shortened the assay time through a one-step stem-loop RT-qPCR method. The platform was able to effectively differentiate between non-small-cell lung cancer (NSCLC) patient and non-patient groups.

In the pursuit of detecting exosomal microRNAs, Zhang et al. innovatively crafted a cutting-edge photothermal ddPCR setup, leveraging the powerful attributes of composite Mxene-based nanocomposites [74]. This state-of-the-art platform has been applied for the accurate and extensive profiling of exosomal microRNA expression within intricate duplex cell samples. The cornerstone of this innovation lies in the effective integration of Mxene nanomaterials with the exterior of magnetic bead surfaces. By employing flow-focusing microfluidic techniques, the system produces hydrogel microcapsules that encapsulate cDNA templates, PCR reagents, and photothermally responsive Mxene nanoparticles, all within their tiny spherical volumes. Utilizing a custom-developed automated near-infrared (NIR) control module, the system facilitates the detection of double-stranded miRNA through a streamlined benchtop procedure, leveraging the adjustable thermal response of the Mxene nanomaterials. This process encompasses reverse transcription and PCR thermocycling steps. The results obtained demonstrated the efficacy of the novel NIR-triggered ddPCR platform in distinguishing prostate cancer patients from healthy individuals based on exosomal miRNA. The exosomal miRNA variants, including miRNA-21-5P, miRNA-375-3P, and miRNA-574-3p, were found to be upregulated by 7 to 210 times in the cancer group compared to the control group.

In the detection of exosomal proteins, Lin et al. developed a TRACER platform that utilizes proximity-induced ddPCR and is activated by a double-pair amino coupling agent [75]. This platform facilitates the detection of tumor exosomal PD-L1. The concurrent labeling of PD-L1 and EpCAM using their respective extended double-pair amino coupling reagents enables the differentiation between normal cells and tumor cells. In the presence of a ligation probe and ligase, closed ligation is possible because the two extended aptamers are in close proximity. Subsequent to this, ddPCR can be utilized to quantify the ligation product, which in turn indicates the level of Exo-PD-L1 originating from tumor cells. The ddPCR findings showed a direct relationship between exosome concentration and the PCR product copy number (R^2^ = 0.997), with a calculated LOD of 0.0735 pg/mL. The TRACER method successfully differentiates exosomes originating from cancer patients’ plasma, which exhibit positive Exo-PD-L1 markers, from those of healthy individuals. Furthermore, Figure 6c illustrates that Guan et al. developed a new method called CACBA for diagnosing breast cancer by detecting tumor-associated protein-positive exosomes [71]. CACBA combines the technologies of CRISPR/Cas12a and aptamer chemiluminescence to detect both the total concentration of exosomes and the concentration of specific membrane proteins such as EpCAM and MUC1. By calculating the percentage of exosomes that are positive for specific proteins, it is possible to more accurately reflect the status of the tumor and reduce the interference of individual differences, drugs, and other factors. With high sensitivity, strong specificity, excellent anti-interference ability, and stability, CACBA has demonstrated higher accuracy and robustness than commercial methods in the detection of clinical samples. Experimental results show that CACBA can effectively differentiate between breast cancer patients and healthy people, and its accuracy and robustness are better than those of traditional tumor marker assays. CACBA provides a new method for the diagnosis of breast cancer, which is expected to improve the accuracy and early diagnosis, can be extended to the diagnosis of other cancers, and has broad application prospects. Additionally, Zhao et al. produced a biochip based on quantum Weak Value Amplification (WVA) technology for the highly sensitive and specific detection and identification of breast cancer exosomes [76]. The core technology of the chip is WVA, which can amplify weak signal changes through specific optical circuit design and signal processing methods, thus improving detection sensitivity. In addition, the chip uses a zirconium ionization biochip to capture exosomes and enhance the detection signal with aptamer-modified gold nanoparticles (AuNPs). The zirconium ionization biochip utilizes the specific interaction between zirconium dioxide and the phosphate groups on the lipid bilayer of exosomes to capture exosomes quickly and efficiently. The aptamer-modified AuNPs can specifically recognize proteins on exosomes, further amplifying the detection signal and improving the specificity of the assay. The study shows that this detection system has high sensitivity and specificity and can rapidly detect exosomes at low concentrations. It is expected to be an effective tool for the early diagnosis, efficacy monitoring, and prognostic assessment of breast cancer.

The combined detection of multiple exosomal inclusions based on microfluidic sensing devices has been partially reported. For example, as shown in Figure 6d, Xu et al. developed a novel microfluidic platform called ddSEE for the simultaneous detection of proteins and miRNA at the level of individual extracellular vesicles (EVs) [72]. ddSEE leverages a dual digital CRISPR-Cas system for the detection of extracellular vesicle (EV) surface proteins and internal miRNA. Specifically, CRISPR-Cas12a is utilized to identify EV surface proteins, with the assistance of antibody–DNA conjugates that translate protein detection into DNA signals. Additionally, CRISPR-Cas13a is employed for the detection of miRNA within EVs, a process facilitated by encapsulating the CRISPR-Cas13a reagents within liposomes. The subsequent fusion of these liposomes with EVs enables the delivery of the reagents into the vesicles, thereby allowing for the detection of intravesicular miRNA. The authors created a microfluidic device equipped with 188,000 microscopic wells, transforming the CRISPR-Cas technology into a digital platform for the precise enumeration of extracellular vesicles. By applying ddSEE to breast cancer diagnosis and detecting both PD-L1 protein and miR-21 in EVs, they were able to effectively differentiate between breast cancer patients and healthy volunteers with a diagnostic accuracy of 92%. Compared with detecting PD-L1 or miR-21 alone, the simultaneous detection of the two markers can significantly improve the accuracy and specificity of diagnosis.

#### 3.1.5. Other Biomarkers

In addition to the aforementioned biomarkers such as CTCs, ctDNA, miRNA, and exosomes, proteins and metabolites also serve as crucial targets in liquid biopsies for cancer. The application of proteins and metabolites in liquid biopsies can provide new insights into the biological mechanisms of cancer by uncovering anomalies in the protein expression profiles and metabolic alterations of tumor cells. Furthermore, this approach can guide the selection of personalized treatment strategies and the facilitate real-time monitoring of therapeutic efficacy in clinical practice. Cai et al. devised a novel single-cell protein sequencing method called DMF-Protein-seq to gain a more comprehensive understanding of cellular phenotypes and life activities [77]. Using digital microfluidics (DMF) and DNA-tagged antibodies, the method converts the protein information of individual cells into DNA sequences through a “split-and-merge” strategy and labels each cell with a unique cellular barcode. DMF-Protein-seq is capable of detecting protein expression in thousands of single cells simultaneously and accurately identifying clusters of cells, showing great potential for cell sorting and drug monitoring. With higher sensitivity and throughput than conventional flow cytometry and the ability to detect a wider range of protein types, this method provides a new tool for single-cell biology and biomedical applications and contributes to a deeper understanding of cellular heterogeneity, disease mechanisms, and drug mechanisms of action. Meanwhile, to explore the behavior heterogeneity of CTCs during metastasis and its relationship with metabolic profiles, Hou et al. invented an integrated microfluidic system that simulates the tumor metastasis process and combines it with single-cell mass spectrometry to analyze the metabolic profiles of individual CTCs [78]. They used spherical HCT116 cells as a model and investigated the retention patterns of CTCs from different spheroidal body regions under simulated vascular shear flow. It was found that there was heterogeneity in the retention behavior of CTCs, with some CTCs showing greater retention capacity, while others were more prone to detachment from the vessel wall. Through single-cell metabolomics analysis, the researchers found that subpopulations of CTCs with different retention capacities had different metabolic profiles. Among them, the subpopulation of CTCs with greater retention capacity showed the upregulation of metastasis-related metabolic pathways, such as amino acid metabolism, the urea cycle, and the Warburg effect. In addition, the researchers investigated the effects of the antitumor drug 5-fluorouracil on the metastatic behavior and metabolism of CTCs. The results showed that 5-fluorouracil could inhibit metastasis by reducing the proportion of highly metastatic cells in the spherical shape and alter the metabolic profile of CTCs. This study reveals the molecular mechanisms underlying the metastatic behavior of CTCs and provides new ideas for the development of new anti-metastatic therapeutic strategies.

### 3.2. Pathogenic Bacteria Assessment

With the ever-changing global health and safety situation, the prompt and delicate identification of pathogenic bacteria is vital for the prevention and management of diseases, as well as for ensuring public health security. Microfluidic biosensors, with their miniaturization, integration, automation, and high throughput, offer new solutions for pathogenic bacteria detection. These sensors can be used in multiple steps such as sample processing, target recognition, signal amplification, and detection to achieve the rapid, highly sensitive, and specific detection of specific pathogenic bacteria. In the future, with the continuous development of novel recognition elements, chip design, and artificial intelligence technology, microfluidic biosensors are expected to play a greater role in the field of pathogenic bacteria detection and make important contributions to disease prevention and control and public health safety. A brief summary of this next section is shown in Table 3.

#### 3.2.1. Pathogenic Bacteria Identification

Microfluidic biosensors have received a lot of attention for their potential applications in the detection of pathogenic bacteria. The use of specific capture probes in microfluidic biosensors can improve the selectivity of target bacteria detection in complex samples. The identification of specific target bacteria is required prior to pathogenic bacteria detection. Various strategies have been applied in microfluidic biosensors for the recognition and capture of pathogenic bacteria. They mainly include recognition strategies based on antibody–antigen interactions, nucleic acid aptamer binding, phage biorecognition, and antibiotic/antimicrobial peptide recognition.

**Figure 7:**
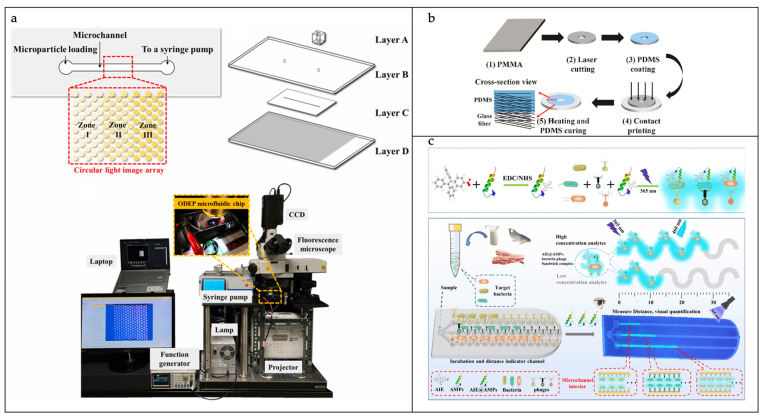
Microfluidic biosensors for pathogenic bacteria identification. (**a**) An illustration depicting the top-view layout and laminated structure of the ODEP microfluidic chip, along with an image of the complete experimental setup (Layer A is a fabricated PDMS connector, Layer B is an indium–tin–oxide (ITO)-coated glass substrate, Layer C is a double-sided adhesive tape with a hollow microchannel, and Layer D is ITO-coated glass with a photoconductive material layer). Reproduced with permission from Ref. [81]. Copyright 2023 Sensors and Actuators B: Chemical. (**b**) The procedure for creating PDMS patterns on a paper base. Reproduced with permission from Ref. [84]. Copyright 2024 Talanta. (**c**) The chip for fluorescence-labeled distance measurement, facilitating the visual identification of pathogens. Reproduced with permission from Ref. [85]. Copyright 2025 Sensors and Actuators B: Chemical.

The antibody–antigen interaction strategy is the most widely used strategy in microfluidic biosensors for achieving the specific recognition of target bacteria in various complex samples [79,80]. In most microfluidic biosensors, whole bacteria/cells are captured by various bacterial/extracellular receptors to form a sandwich structure without the need to lyse the cells for further amplification operations. In the process of bacterial capture, immunomagnetic beads (IMBs) formed by magnetic nanoparticles (MNPs), modified with antibodies, are usually used to target bacteria. These are easy to clean, separate, and enrich under the action of an external magnetic field. Kim et al. achieved the detection of *Vibrio parahaemolyticus* by using IMBs and antibody-modified, functionalized europium fluorescent-labeled particles [80]. By utilizing antibody–antigen interactions, the target bacteria were successfully trapped, leading to the acquisition of a fluorescence signal from the resulting immune complex. This method allowed for the precise quantification of *Vibrio parahaemolyticus* at the individual cell level within a 0.1 mL sample of broth culture. As shown in Figure 7a, Chu et al. engineered an innovative microfluidic device that harnesses optically induced dielectric electrophoresis (ODEP) technology, enabling the swift extraction and purification of viable bacteria directly from blood [81]. The system’s initial function is the removal of the majority of blood cells, a process that employs an immunomagnetic bead separation technique. Subsequent to this, the ODEP microfluidic system is utilized for the purpose of further isolating and purifying live bacteria, a process that is contingent upon the disparity in size between bacteria and blood cells. The ODEP microfluidic system incorporates an integrated dynamic circular photoimage array that directs blood cells to the side channel and collects bacteria downstream of the main channel. It has been demonstrated that this method enables the collection of live bacteria with a purity level ranging from 90.5% to 99.2%, without any observed impact on the functionality of concomitant cells.

Antibodies have good specificity and selectivity, but are less stable and usually more expensive. In recent years, nucleic acid aptamers have been extensively utilized in target recognition applications. In comparison with antibodies, nucleic acid aptamers are more straightforward to synthesize and alter, exhibit higher purity, have a lower molecular mass (~12–30 kDa), are less expensive, demonstrate enhanced resistance to temperature and pH changes, and are easier to store [82]. Li et al. employed a strategy of in situ capture of RCA (cRCA), modifying the inner surfaces of the microfluidic channels with the cRCA product to capture the aptamer in a repetitive tandem with a cell-specific aptamer of *E. coli* O157:H7 [83]. The cRCA product was used to modify the inner surface of the microfluidic channel to repeatedly capture *E. coli* O157:H7 cell-specific aptamers in tandem. In comparison with the single-aptamer recognition method, this approach resulted in a substantial enhancement of the capture efficiency of the target bacteria over a wide flow range. Furthermore, the capture performance in various food matrices (iced tea, bottled water, orange juice, and milk) was analogous to that in phosphate-buffered saline (PBS). Figure 7b shows that Zhu et al. developed a novel pumpless paper/PDMS hybrid microfluidic chip for bacterial concentration assessment and rapid detection [84]. The chip consists of a PDMS reservoir, a hydrophilic paper substrate, microspheres encapsulated with aptamers, and an ultra-absorbent resin, with the PDMS reservoir used to store the sample solution, the paper substrate used for pump-free water transport, the microspheres used to capture the target bacteria, and the ultra-absorbent resin used to maintain a continuous flow of water in the chip. They successfully applied the microchip to detect Pseudomonas aeruginosa in water and diluted milk with a detection limit as low as 10 CFU/mL.

Phages can attack specific bacteria and use their metabolic mechanisms to infect them, and this inherent specificity in interacting with host bacteria can further serve as a specific recognition element for pathogens [86]. As shown in Figure 7c, Liu et al. developed an innovative multi-lane microfluidic chip (D-chip) that the enables swift, concurrent, and visible identification of an extensive array of pathogens [85]. The chip is based on a fluorescence distance readout mode and uses phage and aggregation-induced luminescence fluorophore-labeled antimicrobial peptide (AIE@AMP) coding tags to achieve the specific capture and quantitative analysis of the target bacteria. The D-chip is equipped with three serpentine-shaped channels, pre-coated with three distinct phages, each designed for the selective trapping of one of three target bacterial species. Upon the introduction of the sample into the chip, the various target bacteria are ensnared by their respective channels and transported along the direction of the fluid flow. Subsequently, the AIE@AMPs signaling tag binds to the captured bacteria and enhances their luminescence intensity. By measuring the distance of the corresponding luminescent channels, qualitative and quantitative analyses of different bacterial species can be performed. Under optimized conditions, the D-chip was successfully applied to detect *E. coli* O157:H7 and two *Salmonella* strains with a low detection limit, a wide linear range, a short detection time, and good specificity and stability.

Antibiotics can interact specifically with the cell wall of bacteria and thus be used as capture probes for pathogenic bacteria. The carbonyl group of vancomycin has been demonstrated to bind to the amine group of peptidoglycan in the bacterial cell wall, thus forming strong hydrogen bonds, which has led to the effective utilization of vancomycin in the capture of both Gram-positive (GPB) and Gram-negative (GNB) bacteria. In the field of microbiology, Kim et al. pioneered a significant advancement by integrating dual-antibiotic-conjugated graphene micromechanical field-effect transistors with a microfluidic chip [87]. This novel technique has paved the way for a compact instrument that enables the in situ identification of both Gram-positive and Gram-negative bacterial strains in various samples. The process of pinpointing the target microorganisms is founded on the interactions of charges or chemical entities between the biosensor and the target bacteria. The device boasts a detection sensitivity down to 100 CFU/mL, with an achievable range of 1–9 CFU/mL.

In addition to the above, antimicrobial peptides, DNA enzymes, and lectins have also been applied to bacterial detection. Qiao et al. studied highly stable and low-cost antimicrobial peptides [88]; Ma et al. summarized the application of DNA enzymes in the detection of pathogenic bacteria, where DNA enzymes were used as either recognition elements (RNA-cleaving DNA enzymes) or reporter elements (peroxisome-mimetic properties of DNA enzymes) [89]; Mi et al. provided a comprehensive overview of the potential application of lectins as a target recognition probe in bacterial detection, a field that holds considerable promise [90]. The strategies for bacterial recognition outlined in this study can provide a basis for developing microfluidic biosensors.

#### 3.2.2. Pathogenic Bacteria Isolation

A significant number of microfluidic biosensors are designed to detect bacteria in such a manner that the identification of the target bacteria and the output of the signal are performed concurrently. However, in certain instances, it is imperative to undertake the separation of target bacteria after identification, particularly in the context of complex samples. For the separation of target bacteria, methods based on MNPs, inertia, and centrifugal forces have been applied to microfluidic biosensors.

**Figure 8:**
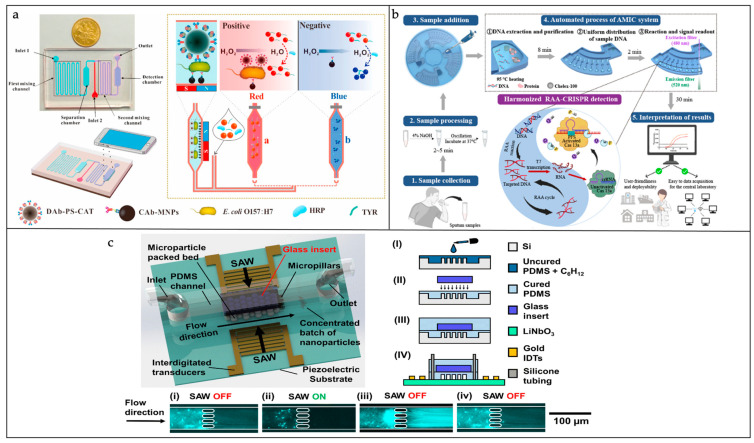
Microfluidic biosensors used for pathogenic bacteria isolation. (**a**) The proposed biosensor uses AuNP clumping and smartphone imaging for the quick detection of *E. coli* O157:H7. Reproduced with permission from Ref. [94]. Copyright 2019 Biosensors and Bioelectronics. (**b**) The AMIC system’s process and the streamlined RAA-CRISPR detection design. Reproduced with permission from Ref. [98]. Copyright 2024 Analytical Chemistry. (**c**) A diagram and the creation of the ultrasonic nanofiltration device. Reproduced with permission from Ref. [99]. Copyright 2023 Lab on a Chip.

The separation technology of MNPs is widely used in microfluidic biosensors. MNP-modified recognition probe molecules can capture the target bacteria, and it is easy to isolate, wash, and enrich the target bacteria under an applied magnetic field [91,92]. Lin’s team reported a series of experiments on the detection of foodborne pathogens using MNPs [93,94,95,96]. The antibody-modified MNPs targeting *E. coli* O157:H7 formed immunomagnetic beads, while the horseradish peroxidase–polystyrene microspheres (PS-CAT) were used to modify the secondary antibodies. This led to the formation of a sandwich-like complex of immunologically coated bacteria, which, via antibody-mediated recognition, trapped and concentrated the target bacteria when subjected to an applied external magnetic field (Figure 8a) [94]. Using the same strategy, the detection of *Salmonella* in food samples was performed [93].

The technique of inertial microfluidics has demonstrated efficacy in the rapid and efficient sorting of cells by size. By engineering microfluidic devices that exploit the size discrepancy between bacterial cells and blood cells, it is possible to isolate and examine bacteria from blood specimens without relying on magnetic field assistance. Lu et al. crafted an unconventional microfluidic channel to amplify the effect of inertial lift, embedded microcubes that would periodically contract along a series of repetitively curved cellular configurations, and achieved the separation of 5.5 μm particles from 6.0 μm particles with purity higher than 92% and greater than 80% recovery. They obtained submicron resolution (submicron) and greater than 80% recovery in a size-based inertial sorting study [97]. A purity of more than 92% and a recovery of more than 80% were achieved with submicron resolution (i.e., 0.5 μm) in a size-based inertial sorting exercise. The purification of two *Candida* species (*Candida albicans* and *Candida smoothii*) from *Candida*-added blood samples using this inertial sorting device enhances the molecular diagnosis of blood *Candida* infections compared to commonly used centrifugal purification methods.

In recent years, disk-based microfluidics, which use centrifugal forces to drive fluid motion, have attracted a great deal of attention in the field of clinical diagnostic bacterial detection. These chips automate sample capture, separation, washing, and reaction by adjusting parameters such as centrifugal angular and linear velocities, microchannel size, and valve design. Wang et al. developed a centrifugal microfluidic chip with eight functional chambers, three siphon valves, and a chitosan-modified PDMS channel for *Salmonella typhimurium* capture, washing, elution, amplification, and detection [100]. As shown in Figure 8b, Xiang et al. designed a fully automated bacterial respiratory infection diagnostic system [98]. The automatic microfluidic diagnostic (AMIC) system is a fully automated diagnostic system for bacterial respiratory infections, automating the process from sample preparation to detection by systematically regulating the sample lysis procedure, the on-chip transcription process, and the lyophilization reagents. Xiao et al. proposed a multichannel molecular diagnostic system, integrating bacterial sample lysis, nucleic acid capture and washout, fluid switching, and a LAMP assay, that combines a simplified design of active valves and the easy fabrication of passive valves [101].

Depending on the size difference between bacteria and blood cells, they can also be separated using acoustic techniques. Ang et al. designed a novel microfluidic device in which a piece of glass was embedded in the top of the PDMS microfluidic channel to form an acoustic reflective surface to improve energy retention and allow higher flow rates (Figure 8c) [99]. The device utilizes acoustic wave-activated nanosieve technology to enrich nanoparticles through microsphere resonance. The experimental data reveal that the apparatus enhances the flow rate by 30 times compared to a version lacking the glass insert, all while preserving a capture efficiency of over 90%. Both numerical simulations and analytical modeling corroborate the glass insert’s effectiveness in boosting energy retention. The apparatus is poised for application in diverse diagnostic settings, including the concentration of exosomes and the isolation of bacteria in assay protocols.

#### 3.2.3. Pathogenic Bacteria Detection

The optical detection methods employed in microfluidic biosensors encompass a range of techniques, including fluorescence, chemiluminescence, colorimetry, and Raman spectroscopy. Among these methods, fluorescence and colorimetry are the most prevalent.

Fluorescent analytical methods with a wide linear response range and high sensitivity are widely used in microfluidic biosensors for the detection of bacteria. Ahn et al. developed a recombinant enzyme polymerase amplification (RPA)-based paper microarray for the single-step detection of a variety of foodborne pathogens [102]. Reagents and fluorescent probes tailored for RPA were added to the reaction compartment, which was subsequently left to air dry. The RPA process was triggered at a constant temperature of 37 °C, lasting for a total of 20 min. Once the reaction compartment was filled with the RPA mixture and fluorescent probes and completely dried, the pathogen DNA was injected into the chip via its designated injection aperture. Thereafter, the RPA reaction was initiated for a further 20 min at a temperature of 37 °C. The chip is capable of the simultaneous detection of *Escherichia coli*, *Staphylococcus aureus*, and *Salmonella* with an LOD of 10^2^ CFU/mL.

**Figure 9:**
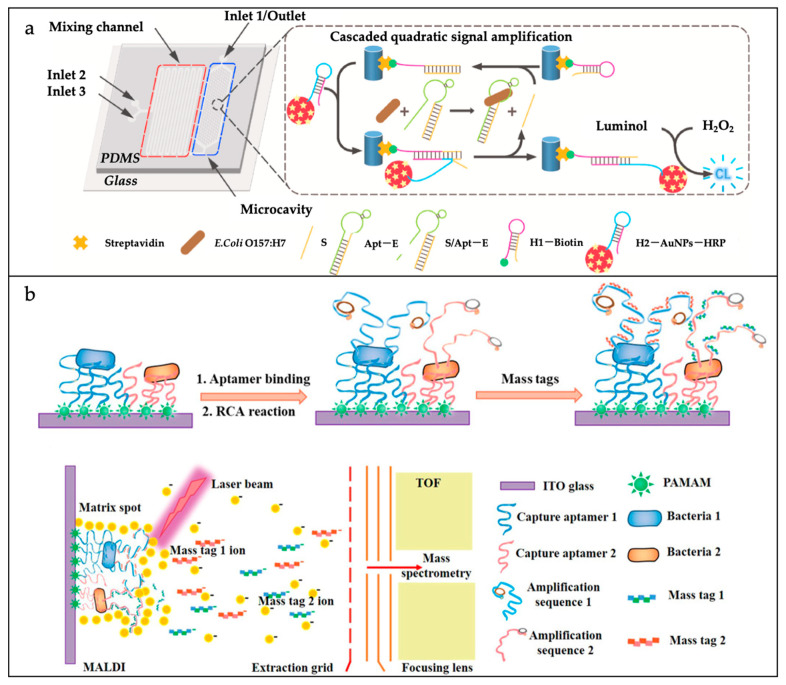
Microfluidic biosensors for pathogenic bacteria detection. (**a**) An illustration of the MCL biosensor using a signal amplification strategy for *E. coli* O157:H7 detection. Reproduced with permission from Ref. [103]. Copyright 2022 Biosensors and Bioelectronics. (**b**) A guide to using mass tags for bacterial identification via MALDI-TOF MS. Reproduced with permission from Ref. [104]. Copyright 2022 Analytical Chemistry.

Chemiluminescence eliminates the need for an excitation light source, avoids the background interference caused by the excitation light source, has low noise, is highly sensitive and selective, and is commonly used in microanalysis. Due to these advantages, microfluidic sensors that rely on chemiluminescence are employed for bacterial detection. As shown in Figure 9a, Sun et al. created a microfluidic chemiluminescence biosensor that incorporates various signal amplification techniques to enable the swift and highly sensitive identification of *E. coli* O157:H7 [103]. Their designed chip consists of a serpentine-shaped mixing channel at the upstream end and a boat-shaped microchamber at the downstream end. The mixing channel is used to efficiently mix luminal and hydrogen peroxide, and the microchamber is used to immobilize biomolecules and perform CHA amplification reactions and chemiluminescence (CL) detection. The targeted bacteria attach to the aptamer, causing the release of a promoter chain that initiates an enzymatic CHA amplification process involving H1 and H2, leading to the formation of H1/H2 bilayers. Concurrently, Au NPs-HRP are fixed onto the chip, where they facilitate the chemiluminescent reaction between lumefantrine and hydrogen peroxide, producing a measurable light signal. The microfluidic biosensor has a low LOD of 130 CFU/mL for *E. coli* O157:H7, a wide dynamic range, and a short analysis time (~1.5 h).

Colorimetric assays are widely used because the results can be observed directly with the naked eye, and the core of the assay is the conversion of biometric signals into color change signals. The use of enzyme-catalyzed chromogenic substrates to produce color signals is a popular form of colorimetric assay, and the TMB-catalyzed system is a commonly used system for visual detection. Wang et al. successfully identified *E. coli* and multidrug-resistant *Staphylococcus aureus* by using a two-adaptor system to recognize the target bacteria to form a sandwich structure, and the horseradish peroxidase (HRP)-catalyzed TMB system showed blue color signals, which could be detected within 35 min [105]. In addition, the sensitivity of the assay can be enhanced through the isothermal amplification of nucleic acids using methods like HCR and LAMP [106]. Trinh et al. constructed a flexible, visual detection microfluidic platform capable of identifying a variety of foodborne pathogens [107]. The device is coated with 2-hydroxyethyl agarose to encapsulate the LAMP reagents, maintaining their efficacy for a minimum of 45 days. The paper’s foldable design facilitates the movement of reagents from the amplification reaction compartment to the detection chamber, employing a straightforward colorimetric method for the immediate visual interpretation of the assay results.

SERS has emerged as an effective technique for bacterial identification due to its non-destructive detection of multi-component samples, on-line analysis, and direct access to molecular fingerprinting information. Witkovska et al. detected *Porphyromonas gingivalis* and *Porphyromonas actinomycetemcomitans* aggregates using SERS on microfluidic microarrays with the use of silver-plated magnetic nanoparticles (Fe_2_O_3_@AgNPs) [108]. The target bacteria were adsorbed and thus isolated from complex samples and subsequently transferred to a Si/Ag SERS detection platform for detection. Zhuang et al. developed a novel microfluidic paper-based assay device (μPAD) for the rapid detection of disease-causing bacteria, e.g., *salmonella*, in food products [36]. The device combines CRISPR/Cas12a and surface-enhanced Raman scattering (SERS) technologies, using CRISPR RNA (crRNA) to guide the Cas12a protein to recognize and cleave the target DNA, and taking advantage of the surface-enhanced Raman scattering of precious metal nanostructures to achieve the highly sensitive and selective detection of trace substances with an LOD of about 3–4 CFU/mL. The platform utilizes capillary-driven liquid flow to integrate sample handling, reaction, and detection.

Electrochemical biosensors are suitable for the rapid in situ detection of pathogens without the need for the pre-treatment of various sample matrices. Jiang et al. developed cloth-based microfluidic electrochemical sensors (ECDSs) for the first time, modifying CdTe quantum dots/nanocomposites (CdTe-MWCNTs) on cloth-based microfluidic chips prepared by carbon ink and solid wax screen-printing techniques to enhance ECL signals. They also performed a double linear hybridization chain reaction (DL-HCR), combined with simple enzymatic digestion, to detect *Lactobacillus monocytogenes* in milk samples, with an LOD of about 1.64 × 10^4^ CFU/mL [109]. Hou et al. created an innovative microfluidic biosensor designed for the highly sensitive and swift detection of *Salmonella* spp. [110]. The biosensor utilizes a self-assembled MNP chain to separate *Salmonella* spp. in a continuous flow from larger-volume samples. The highly sensitive detection of *Salmonella* was performed using linear scanning voltammetry. The magnetically responsive bacteria were mixed with urease-coated AuNPs within a three-dimensional spiral conduit and then introduced into a chip equipped with an array of thin-film Ag/AgCl reference electrodes to perform linear scanning voltametric analysis of *Salmonella*. This process allows for a detection duration of under one hour and achieves an LOD as low as 10 CFU/mL.

The deployment of matrix-assisted laser desorption/ionization time-of-flight (MALDI-TOF) mass spectrometry is seen as a prompt and trustworthy approach for the recognition of bacterial entities. A unique library of standard profiles has been constructed for common foodborne pathogenic bacteria by utilizing their distinct fingerprint mass spectra. The identification process involves the comparison of the profiles with those stored within the database. In addition, Li et al. developed a rapid method for the identification of bacteria present in urine samples. This encompasses capturing bacteria on a microfluidic chip and then employing MALDI-TOF MS to detect them [111]. This method successfully detected bacterial concentrations ranging from 10^6^ CFU/mL to 10^3^ CFU/mL in urine samples following an incubation period of four hours. The concentration of bacteria in urine samples was 10^6^ CFU/mL, and 10^3^ CFU/mL was detected after 4 h of incubation; however, this method can only identify the bacteria and not quantitatively analyze them, relying on the database of standard profiles. To address these problems, Li et al. proposed a new mass-label-mediated strategy for the simultaneous detection of multiple bacteria by MALDI-TOF MS (Figure 9b) [104]. Carboxy-functionalized polyamides (PAMAM) were introduced to modify the aptamers of target bacteria with regard to the wells at different positions on the target plate. When the target bacteria appeared, the target bacteria were captured on the target plate, and the excess non-target bacteria and impurities were washed away. Then, the aptamers connected with the RCA primer were added to bind specifically to the target bacteria, and RCA amplification was carried out after washing. After amplification, a DNA sequence complementary to the amplified sequence is added as a mass tag, washed, and laser-irradiated to release the mass tag, which is ionized for mass spectrometry detection. This method converts bacterial detection into DNA mass tag analysis, eliminating the reliance on microbial mass spectrometry databases and enabling the simultaneous detection of multiple target bacteria.

### 3.3. Point-of-Care Testing (POCT)

The steady increase in the prevalence of inflammation, infectious diseases, and chronic conditions seen globally has highlighted the urgent requirement for innovative diagnostic approaches. While conventional diagnostic techniques are effective, they frequently involve invasive processes, demand complex machinery, and may not be readily accessible when needed. This has ignited a strong interest in the advancement of POCT technologies, especially microfluidic biosensing platforms, which present swift, sensitive, and precise diagnostic capabilities. This section is committed to the in-depth analysis of these platforms, with an emphasis on the recent advancements in the realms of inflammatory disease, infectious disease, and chronic condition diagnosis. By examining these case studies, we intend to underscore the revolutionary role that microfluidic biosensors are playing in the progression of POCT, heralding a future where prompt and exact medical interventions are more accessible. An overview of the following section is provided in Table 4.

#### 3.3.1. Inflammation Biomarker Point-of-Care Testing

**Figure 10:**
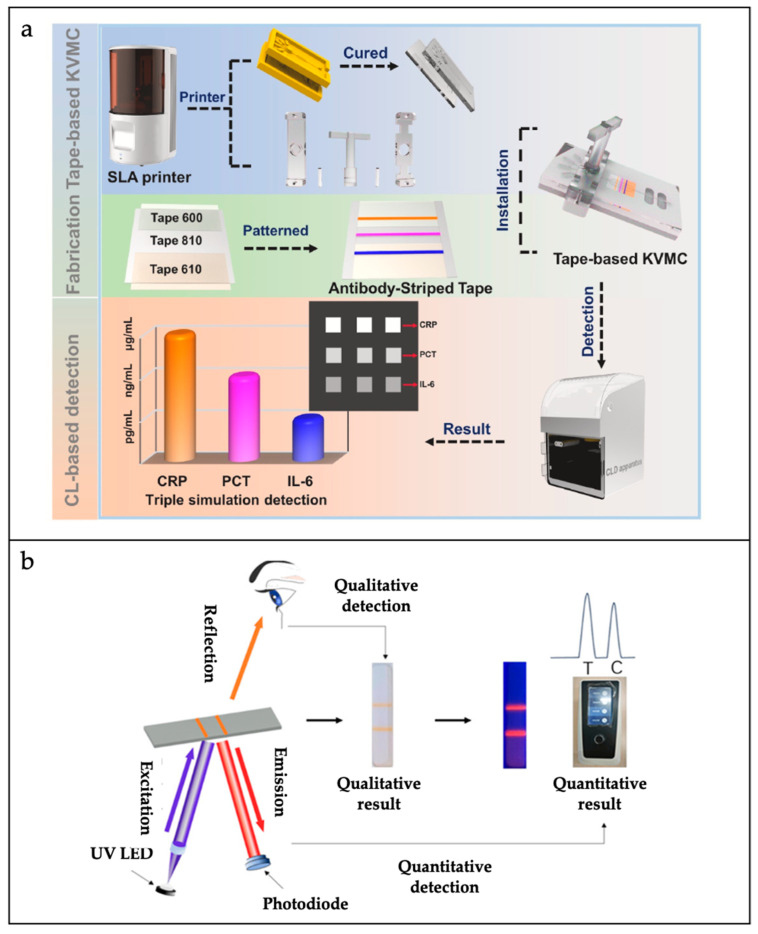
Microfluidic biosensors for inflammation biomarker POCT. (**a**) A schematic of a 3D-printed Tape-based KVMC system for versatile immunoassays with tunable sensitivity. Reproduced with permission from Ref. [112]. Copyright 2022 Biosensors and Bioelectronics. (**b**) The principles of detection and representative outcomes for dual-modal lateral flow immunoassays with colorimetric and fluorescent readouts. Reproduced with permission from Ref. [113]. Copyright 2024 Analytical Chemistry.

Figure 10a illustrates the development of a tape-integrated self-designed microfluidic chip for simultaneous detection via point-of-care immunoassays, termed Tape-based KVMC, by Yin et al. [112]. The KVMC molds were fabricated using 3D printing technology and combined with 3M adhesive tape as the antibody fixation layer. Three types of 3M tapes (Tape 610, Tape 810, Tape 600) with different adsorption characteristics were used to immobilize antibodies for CRP, PCT and IL-6, allowing the dynamic adjustment of the detection range. The simultaneous detection of CRP, PCT and IL-6 is achieved by rotating a key valve to control the flow sequence of different reagents, with the signal being detected using chemiluminescence. The results showed that the Tape-based KVMC was able to dynamically detect CRP, PCT, and IL-6 in the range of pg mL^−1^ to μg mL^−1^ with high sensitivity, high selectivity, and good reproducibility. In addition, the results of Tape-based KVMC for CRP, PCT, and IL-6 in clinical serum samples were consistent with those of ELISA kits, indicating that it has a promising future for clinical application. As shown in Figure 10b, Fan et al. successfully engineered an aggregation-induced luminescent nanobead (AIENB)-based dual-readout lateral flow immunoassay (LFA) for the expedited and sensitive detection of CRP in clinical specimens [113]. The AIENB-LFA was fabricated through the encapsulation of red AIE luminophores possessing both fluorescent and chromogenic attributes within polymer nanobeads, utilizing an emulsification–solvent evaporation technique. This assay demonstrates qualitative detection capabilities for CRP at a concentration as low as 8.0 mg/L under naked-eye visualization, and it can quantitatively measure high-sensitivity C-reactive protein (hs-CRP) in the fluorescence mode with an LOD of 0.16 mg/L. The performance of the AIENB-LFA is on par with the immunoturbidimetric assay, exhibiting equivalent detection limits and a strong correlation with the immunoturbidimetric assay. The AIENB-LFA developed in this investigation features dual reading modalities—fluorescence and color development—along with high sensitivity, quantification capabilities, and straightforward operation, and offering a novel approach for the rapid and sensitive detection of CRP and hs-CRP. It has significant potential for widespread application. Boonkaew et al. developed a portable electrochemical biosensor based on NFC and nanobodies for the detection of the inflammatory biomarker CRP [114]. To overcome the limitations of conventional POCT devices in terms of sensitivity, selectivity and storage stability, they used phage display technology to screen nanobodies against CRP and immobilized them on screen-printed graphene electrodes. CRP was detected by chrono coulometry (CC) and wireless data transmission and smartphone control were achieved using NFC technology. The results showed that the sensor has high sensitivity, good specificity, and portability, and can accurately detect CRP in clinical samples without sample pretreatment. It has promising applications in the diagnosis of inflammatory diseases, infectious diseases, and cardiovascular diseases.

A microfluidic biosensor-based POCT inflammation detection device can directly detect inflammatory markers and assess the degree of inflammation using biochemical indicators, in addition to making morphological observations. Yang et al. developed a portable micro-imaging system for the rapid detection of vaginal inflammation in women at home [115]. They designed a self-absorbing microfluidic chip for automatic sample collection and adequate mixing, and developed an optical system adapted to smartphones with 37× magnification and a focusing range of 4 mm to 6 mm. Meanwhile, they designed a mobile phone app to accurately identify cocci and determine the degree of inflammation. Tested on simulated and clinical samples, the system demonstrated high resolution, high accuracy, and rapid detection. The system provides a simple and accurate method to allow women to detect vaginal inflammation, which helps to raise women’s awareness of their own health and to detect and treat inflammation in a timely manner. It also provides a new technological means for the rapid diagnosis and treatment of vaginal inflammation.

There have also been reports of combining microfluidic POCT devices use for inflammation detection with wearable devices. Tu et al. developed a wearable biosensor patch called the InflaStat in response to health problems caused by chronic inflammation and acute inflammatory responses, as well as the limitations of traditional inflammation monitoring methods [116]. The sensor integrates an electrodialysis sweat extraction module, microfluidic channels, and graphene-based sensor arrays to enable the real-time, non-invasive monitoring of CRP levels in sweat. By integrating pH, ionic strength, and temperature sensors for real-time calibration, the technology effectively eliminates errors associated with inter-individual differences in sample matrices and enables the highly sensitive electrochemical detection of CRP. Strong correlations between sweat CRP levels and blood CRP levels were demonstrated in healthy subjects and patients with heart failure and acute or previous infections (e.g., COVID-19), suggesting that this technology can be used for non-invasive disease classification, monitoring, and management. This study provides a new solution for non-invasive inflammation monitoring, contributes to chronic disease management and early intervention, and offers new possibilities for personalized medicine and telehealth management.

#### 3.3.2. Infectious Disease Point-of-Care Testing

Malaria, a parasitic illness resulting from *Plasmodium vivax* infection in the human host, necessitates effective diagnostic strategies. The existing diagnostic methods, which require specialized operators for the identification of infected cells, are often incompatible with the resource-poor regions that frequently experience malaria outbreaks [117]. Consequently, there is an urgent requirement for the development of highly sensitive, simple, and safe detection technologies for *Plasmodium* species, particularly ones tailored to the constraints of these underserved areas. Malpartida-Cardenas et al. investigated a novel molecular assay, targeting the *Kelch 13* gene, that is capable of rapidly and specifically detecting *Plasmodium falciparum* malaria by detecting a change in the pH value of the solution through electrochemical methods based on the LAMP reaction [118]. The method uses DNA samples from *P. falciparum* clinical isolates for the first time for quantitative DNA detection via microarrays and further validates the C580Y single-nucleotide polymorphism (SNP) associated with artemisinin-resistant malaria. The results of this study were used for the rapid screening of *P. falciparum* malaria in resource-limited areas. Subsequently, Malpartida-Cardenas et al. designed seven species-specific LAMP assays for *P. falciparum* in humans, and evaluated their performance using synthetic DNA and clinical samples [119]. Furthermore, they conducted a local field trial in Ghana using a handheld Lacewing microfluidic chip platform to assess the potential of this assay for POCT applications. The results show that their design is a promising diagnostic tool for POCT malaria that could help to detect asymptomatic malaria cases, reduce malaria transmission, and improve malaria control strategies.

For bacterial infections caused by prevalent pathogens such as *Escherichia coli*, *Staphylococcus aureus* and *Salmonella*, the conventional detection strategies predominantly involve microbiological culture techniques, which rely on the identification of microorganisms through morphological and biochemical assessments [102]. These approaches are typically laborious and time-intensive, failing to satisfy the demand for expeditious diagnosis in the context of acute infection. In response to this critical challenge, there has been a concerted effort by researchers to advance the development of POCT devices based on microfluidic biosensors. Shang et al. have developed a rapid, accurate, and easy-to-use detection platform for the detection of foodborne bacteria in food samples [120]. They constructed a fully integrated microplatform called FID-MP, which combines nucleic acid extraction, RPA, and signal detection. FID-MP is capable of detecting 8 foodborne bacteria simultaneously, including *Salmonella*, *Staphylococcus aureus*, *Bacillus cereus*, *Pseudomonas aeruginosa*, enterohaemorrhagic *Escherichia coli* O157:H7, *Enterobacter sakazakii*, *Listeria monocytogenes*, and *Vibrio parahaemolyticus*. The FID-MP has the same specificity and sensitivity as a standard real-time RPA reaction with a detection time of less than 60 min. The platform is easy to operate, requires no large equipment or specialized personnel, is portable and scalable, and is expected to be a powerful tool for foodborne bacteria surveillance and for public health incident management.

**Figure 11:**
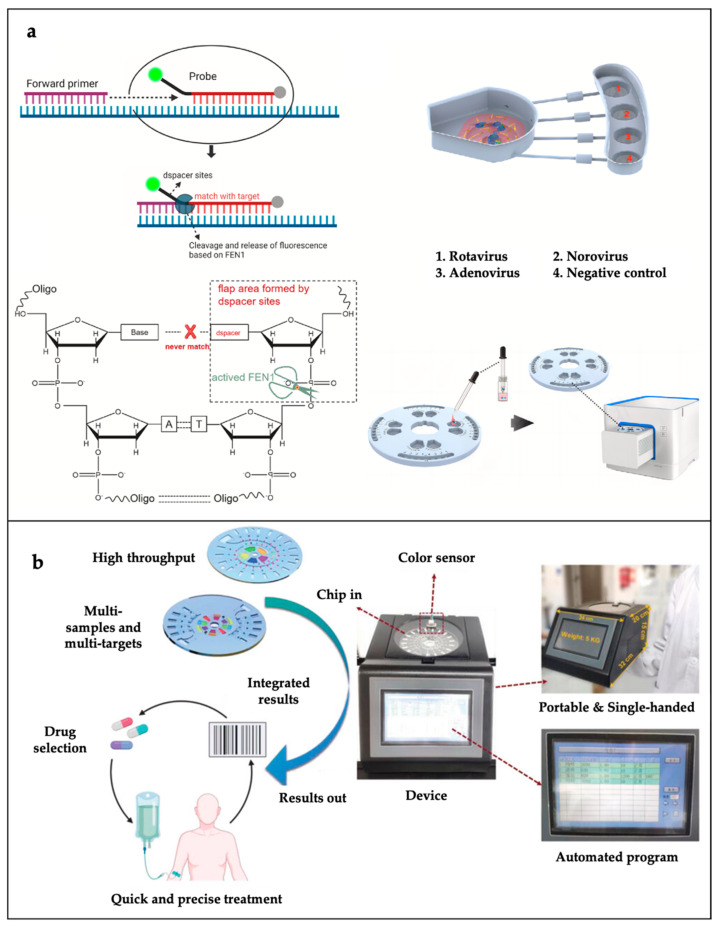
Microfluidic biosensors for POCT infectious disease detection. (**a**) An overview of the detection process and usage steps for the current MFIA setup. Reproduced with permission from Ref. [121]. Copyright 2024 Biosensors and Bioelectronics. (**b**) An illustration of the Dac system for ELISA and its comparative analysis with the conventional method. Reproduced with permission from Ref. [123]. Copyright 2024 Small.

In the research by Ye et al., as shown in Figure 11a, a novel method was proposed for the rapid and simultaneous detection of rotavirus, norovirus, and adenovirus [121]. These viruses are among the most common causes of childhood diarrhea. The novel method, microfluidic-FEN1-assisted isothermal amplification (MFIA), combines the specificity-enhancing properties of the FEN1 enzyme and the adaptability of microfluidic technology. The suggested MFIA technique combines the enhanced specificity attributes of the FEN1 enzyme, a universally applicable dSPACE-altered flap probe, with the versatility of microfluidic platforms, thereby attaining exceptional sensitivity and selectivity for prevalent diarrheal pathogens. Among other things, the FEN1 enzyme recognizes and cleaves dspacer-modified flap structures, releasing fluorescent moieties that generate detectable signals. They developed a microfluidic chip containing 8 independent detection units that can analyze 8 samples simultaneously and use centrifugal force for fluid distribution and reaction. The clinical efficacy of the MFIA was assessed using 150 genuine clinical specimens, demonstrating diagnostic precision on par with the benchmark PCR technique and outperforming antigen-based detection approaches. Moreover, Dai et al. developed an electro-optical multiclassification diagnostic platform capable of accurately identifying disease biomarkers [122]. They constructed a dual-mode multiclassification diagnostic platform (DMDP) that integrates an electrochemiluminescent sensor and a field-effect transistor sensor in a microfluidic chip and uses antibodies and DNA ligands to identify target biomarkers. In clinical validation studies, the DMDP demonstrated near 99% diagnostic accuracy in serum and nasopharyngeal/anal swab samples for tuberculosis (TB), human rhinovirus (HRV), and group B streptococcus (GBS), which was significantly higher than the accuracy of existing bedside diagnostic technologies (77–93%), and the diagnostic time was only 5 min. The development of the DMDP successfully overcame the occasional inaccuracies of existing rapid POCT and achieved the same accuracy as laboratory tests.

Beyond the advancement of streamlined diagnostic procedures for specific infectious ailments, a contingent of investigators is engaged in the development of high-throughput analytical capacities for microfluidic biosensor-integrated POCT systems. In order to address the difficulty of rapid detection during infectious disease outbreaks in resource-limited areas, as shown in Figure 11b, Li et al. developed a high-throughput microfluidic system called Dac, which integrates dual-siphon valves and an air-pressure-balancing structure to achieve the on-demand sequential release of reagents, high-throughput quantitative dispensing, and the collection of solutions, mixing reactions, and waste liquid discharge [123]. In addition, the special protruding structure inside the chip generates microbubbles during the mixing reaction, which further improves the mixing efficiency and analytical sensitivity. The results show that the Dac system can perform 17 independent ELISA reactions simultaneously with only 2% of the reagent consumption of a traditional ELISA, reducing the assay time by more than half. In addition, the Dac system’s multiplexing capability enables the rapid differentiation of multiple diseases and precise therapeutic interventions based on barcode results. Compared to traditional clinical instruments, the Dac system demonstrates high accuracy, specificity, and sensitivity in the classification of clinical inflammation, providing a new solution for rapid and accurate disease diagnosis and treatment in resource-limited areas.

#### 3.3.3. Chronic Disease Point-of-Care Testing

The incidence of chronic diseases has reached epidemic proportions globally, and most chronic diseases (diabetes, hypertension, coronary heart disease, pulmonary and renal diseases, etc.) are usually difficult to cured at once. This, together with the lack of timely medical services in underdeveloped areas, often results in the delayed treatment of patients with chronic diseases, who do not receive timely and effective diagnosis [124], making the rapid on-site detection of chronic diseases particularly important.

**Figure 12:**
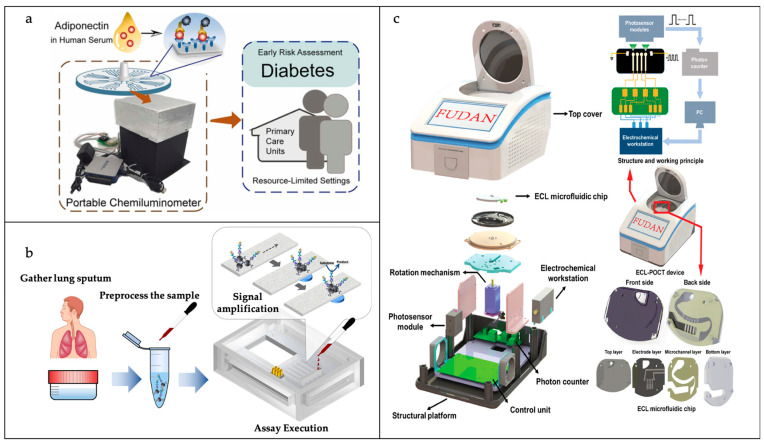
Microfluidic biosensors for POCT chronic disease identification. (**a**) A schematic of the optical detection module of the chemiluminometer. Reproduced with permission from Ref. [126]. Copyright 2023 B Sensors and Actuators B: Chemical. (**b**) An illustration of a portable paper microfluidic electrochemical device for the multiplex quantitative detection of biomarkers in sputum. Reproduced with permission from Ref. [127]. Copyright 2023 ACS Sensors. (**c**) A graph of the ECL-M POCT system. Reproduced with permission from Ref. [128]. Copyright 2024 Advanced Science.

Diabetes mellitus is a metabolic disorder caused by multiple etiological factors, and blood glucose level is an important parameter for monitoring diabetes. The continuous and real-time monitoring of blood glucose is beneficial to diabetes treatment. Jiao et al. engineered a portable, rapid, and low-cost glycated hemoglobin (HbA1c) detection system for the immediate detection of diabetes diagnosis [125]. They designed and fabricated a novel microchip, integrating a micromixer, a passive injector, a packed column, and a detection unit, components which were machined and bonded by micro-milling and laser-welding techniques. The microchip liquid chromatography (LC) system was constructed using a high-pressure syringe pump and a spectrometer, and the separation conditions of HbA1c were optimized. The results showed that the microchip LC system was capable of accurately separating and detecting samples containing four different HbA1c levels within 2 min, with an inaccuracy of less than 3.2%, a coefficient of variation of less than 2.1%, and a correlation coefficient of 0.993. Compared with previously reported microchips, the microchip LC system provides an effective method for the instantaneous detection of HbA1c, and is expected to be used for diabetes mellitus. As shown in Figure 12a, Li et al. developed a polycarbonate-based centrifugal microfluidic chip with 40 parallel detection microchannels and designed a portable chemiluminescence meter to record chemiluminescent signals for the immediate detection of lipocalin [126]. The measurement of lipocalin relies on an immunoassay technique and, with the incorporation of a centrifugal force-driven washing step, the assay process becomes partially automated and user-friendly. This device is affordable, portable, ideal for POCT, and demonstrates both high sensitivity and specificity in detecting lipocalin. It is particularly apt for preliminary diabetes risk evaluation in community health centers and other resource-constrained environments.

Chronic obstructive pulmonary disease (COPD) is the third leading cause of death worldwide, with 20% to 30% of patients experiencing recurrent acute exacerbations (AECOPDs), manifesting as excessive sputum secretion. The immune system of patients with AECOPDs secretes interleukin-8 (IL-8) and tumor necrosis factor-alpha (TNF-alpha), as well as myeloperoxidase (MPO). As shown in Figure 12b, Gutiérrez-Capitán et al. invented a portable, multifunctional microfluidic electrochemical device for the simultaneous quantitative detection of biomarkers associated with acute exacerbations of chronic obstructive pulmonary disease (COPD) (AECOPDs), thereby facilitating the timely detection of AECOPD and improving diagnostic accuracy [127]. The device combines microfluidic paper chip technology, electrochemical sensing, and magnetic nanoparticle-based immunoassays and contains a reusable miniaturized two-electrode array chip and a disposable multi-channel paper fluidic assembly. Sample pretreatment, including biomarker pre-concentration, was performed using magnetic nanoparticles and electrochemical detection was performed by chronoamperometry. The results showed that the device produced calibration curves in buffer and artificial sputum over a range of clinically relevant biomarker concentrations, and the analysis of sputum samples from healthy individuals and AECOPD patients showed statistically significant differences in biomarker concentrations between the two groups. With the advantages of low cost, ease of use, quantitative detection, rapidity and versatility, this device is expected to become a POCT tool for AECOPD diagnosis.

In recent years, acute myocardial infarction (AMI) has emerged as a major disease risk for humans, exerting a substantial burden on global health systems. Troponin (Tn), a protein complex found in cardiac muscle, serves as a highly specific biomarker of myocardial injury. Notably, the concentration of Tn can incrementally rise to detectable nanogram levels in the bloodstream several days prior to the manifestation of acute symptoms, such as chest pain and shortness of breath [129]. This asymptomatic phase provides a critical window for early detection and intervention, which are essential for improving patient outcomes and reducing mortality rates associated with AMI. Yu et al. developed a photothermal biosensing system based on thermal fiber paper [130]. The apparatus enhances the target signal via a series of enzymatic amplification steps, employs paper fibers embedded with thermochromic pigments as sensory material to translate the target signal into a heat response when exposed to laser light, and is designed to create portable, high-capacity devices for the on-site monitoring of target proteins, specifically for Tn detection at the point of care. This method can facilitate the development of thermochromic materials in portable biosensors, but is limited by the determination of only a single cardiac biomarker. To overcome this limitation, as shown in Figure 12c, Xiong et al. developed an ultrasensitive electrochemiluminescence microfluidic system (ECL-M) based on the AC osmotic drive for the rapid simultaneous detection of proteins and miRNA in untreated samples [128]. The ECL-M system combines sandwich immunoassay and surface plasmon resonance (SPR) techniques with the use of cardiac troponin I antigen (cTnI) and miR-499-5p detection models. The system utilizes Ru@SiO_2_ NPs-labeled secondary antibodies as signal probes for the detection of cTnI by the sandwich immunoassay, and employs AuNPs to generate SPR effects to enhance the electrochemiluminescence signal of Ru(bpy)_3_^2+^ for the detection of miR-499-5p. In this method, the detection limits were as low as 2 fg/mL for cTnI and 10 aM for miRNA.

Zhou et al. developed a POCT platform for multiple chronic disease biomarkers in blood using a microfluidic chip and fluorescent oxygen sensitive membrane (OSM) [131]. The sensing platform is characterized by uniform response and sensitive fluorescent signals. First, plasma was separated using a handheld minicentrifuge adapted from a toy fan. Then, signal conversion was achieved in a microfluidic chip using enzymatic oxygen consumption and OSM. Finally, a smartphone was used to acquire the fluorescence signal. The whole process can be performed in the field without the need for specialized instruments. The simple structure of the microfluidic chip (multiple chambers connected by a single channel) eliminates the need for on- or off-chip fluidic control and allows the easy loading and expansion of samples depending on the number of targets. In this POCT platform, a fluorescent OSM is used as the sensor element to convert the concentration of biomarkers into a fluorescent signal, eliminating the peroxidase and chromogenic substrate used in traditional colorimetric analysis, thus reducing reagent costs and facilitating the pre-storage of reagents. Theoretically, targets that are directly or indirectly related to oxygen, such as bilirubin, creatinine and hypoxanthine, can be detected. This low-system-complexity, low-cost, multiplexed POCT device is expected to meet the needs of self-management in patients with chronic diseases. It is also expected to build a “sample-answer” device via the integration of sample preprocessing technology, and a remote monitoring and smart medical device by deeply integrating wireless control, data processing, and data sharing via smartphones.

In addition to the above-mentioned applications in inflammatory, infectious and chronic disease detection, microfluidic biosensor-based POCT devices have applications in many disease detection-related areas, such as tumor detection. Yang et al. developed a novel fluorescent biosensor based on MBene nanosheets for the rapid and sensitive detection of ctDNA, and established a POCT platform based on paper-based microfluidic chips [132]. They successfully synthesized MBene nanosheets by etching and ultrasonic stripping. Then, they revealed MBene’s excellent adsorption capacity for DNA and the recognition of single-stranded DNA (ssDNA) and double-stranded DNA (dsDNA) by density-functional theory (DFT) calculations, and compared them with MXene. Subsequently, they developed a ctDNA assay based on MBene and exonuclease III-assisted fluorescence signal amplification, and achieved the highly sensitive detection of epidermal growth factor (EGFR) 19 del mutation in non-small-cell lung cancer. They designed and prepared a paper-based microfluidic chip containing hybrid channels and delay zones, and verified its performance through numerical simulations and experiments. At the same time, they developed a WeChat app called “ctDNA test” to read the test results on the chip, which realizes the convenience and user-friendliness of POCT.

## 4. Summary: Challenges and Future Perspectives

The emergence of microfluidic biosensors as cutting-edge bio-diagnostic tools has catalyzed a transformative shift in the methodology of disease diagnosis. This technology, characterized by miniaturization, integration, and automation, has led to a paradigmatic change in diagnostic approaches, making them expedient, sensitive, precise, and user-friendly. The utility of microfluidic biosensors is extensive, encompassing applications such as cancer liquid biopsies, pathogenic bacteria detection, and point-of-care testing (POCT).

The categorization of microfluidic biosensors is predicated on the mode of fluidic manipulation and they can be delineated into three primary types: flow channel, digital, and paper-based microfluidic biosensors. Extensive research has confirmed their potential, yet it has also revealed several critical challenges. These include the integration of biosensors with microfluidic platforms, maintaining sensor stability and reproducibility, and the need for cost-effective, disposable chip designs. More specifically, the challenges faced include the following:

Sensitivity and specificity: the quest for higher detection limits and selectivity is paramount for early and accurate disease diagnosis.

Stability and reproducibility: achieving long-term stability and consistent performance is critical for ensuring outcome reliability.

Integration and miniaturization: the drive towards more compact and multifunctional devices through the integration of microfluidic biosensors with other technologies.

Cost-effectiveness: the pursuit of affordable solutions to enable widespread use, particularly in resource-constrained environments.

Clinical validation: robust clinical trials are necessary to confirm the efficacy of microfluidic biosensors in real-world scenarios.

In summation, microfluidic biosensor technology holds immense promise for advancement, although it is confronted by several obstacles. The future portends the development of universally compatible modular systems, which can be customized to fulfill specific diagnostic needs. The amalgamation of 3D printing technology and printed electronics is set to pave the way for the straightforward, economical, and high-throughput production of microfluidic biosensors. The replacement of traditional, cumbersome analytical instruments with more accessible devices, such as smartphones and wearable electronics, for the interpretation and analysis of results represents a significant advancement. Additionally, the application of artificial intelligence algorithms for the deep learning and computational analysis of raw experimental data is anticipated to alleviate the performance pressures on biosensing transducers.

As we look ahead, addressing the current challenges and focusing on the areas for improvement will be key to realizing the full potential of microfluidic biosensors in precision medicine and global health. With continuous technological progression, microfluidic biosensors are primed to play an increasingly vital role in the pantheon of disease diagnostics, significantly contributing to the enhancement of human health.

## Figures and Tables

**Table 1:** Comparison of materials used for the fabrication of microfluidic chips.

Materials		Advantages	Disadvantages
Silicon/Glass-based		High resistance, thermal conductivity, transparency, insulation. Good for electrophoresis, reactions, and cell culture.	High cost. Complex fabrication. Fragile. Limited to specific environments.
Polymer-based	Thermoplastics	Ease of processing and prototyping. Recyclable. Wide range of mechanical properties.	Lower thermal stability. May deform under high temperatures. Potential for leaching of additives.
	Thermosets	High thermal stability. Good chemical resistance. Excellent mechanical properties.	Cannot be remolded or recycled. Lengthy curing process. Potential for shrinkage during curing.
	Elastomers	Biocompatibility. Flexibility. Excellent for soft lithography.	Permeability to certain gasses and solvents. Lower mechanical strength. Potential for swelling in certain solvents.
Hydrogel		Promotes cell adhesion. Suitable for cell culture applications.	Limited strength. Susceptible to degradation.
Paper-based		Low cost. Ease of use. Suitable for point-of-care applications.	Limited sensitivity. Susceptible to environmental factors like humidity and evaporation.
Capillary assembly		3D flow paths. Minimal fluid–wall interaction. Simple assembly.	Manual assembly. Slower production speed. Less efficient for large-scale production.

**Table 2:** An overview of the application of microfluidic biosensors in tumor liquid biopsy.

Elements	Content Description
Detection targets	Circulating tumor cells (CTCs). Circulating tumor DNA (ctDNA). MicroRNA (miRNA). Exosomes. Other biomarkers (e.g., proteins and metabolites).
Purpose	Early diagnosis. Accurate treatment. Prognostic assessment.
Microfluidics applications	The efficient processing and analysis of trace biological samples. Precise isolation, enrichment and detection of cancer-related molecules.
Technical advantages	Enhanced detection sensitivity and specificity. Automated and integrated processes. Reduced sample consumption.
Future directions	Developing novel tumor biomarker recognition elements and innovative signal transduction mechanisms. Enhancing precision in single-cell analysis to more thoroughly resolve tumor cell heterogeneity. Integrating microfluidic biosensors with a range of assays, including genomics, transcriptomics, proteomics, and metabolomics, for comprehensive multimodal analysis of liquid biopsies. Advancing the clinical utility of microfluidic biosensors in tumor liquid biopsy, which includes the creation of universally accessible liquid biopsy devices and assays, as well as the establishment of comprehensive clinical application guidelines.

**Table 3:** An overview of the application of microfluidic biosensors in pathogenic bacteria detection.

Elements	Content Description
Key Technical Features	Rapid identification. High sensitivity. Elevated specificity.
Technology Used	Antibody–antigen interaction. Nucleic acid aptamer binding. Phage biorecognition. Antibiotic/antimicrobial peptide recognition strategies.
Technical Advantages	Higher selectivity and accuracy than traditional methods. Allow specific identification in complex samples. Efficient purification and detection of live pathogenic bacteria.
Future Directions	Exploring more sensitive and specific recognition elements, such as novel antibodies, nucleic acid aptamers, phages, etc., to enhance the accuracy and reliability of pathogenic bacteria detection. Optimizing microfluidic chip design to enhance pathogen isolation efficiency and purity, yielding high-quality samples for subsequent testing and analysis. Developing novel pathogen detection methods to increase the sensitivity and specificity of pathogen detection. Utilizing the deep learning and computational analysis of data generated by microfluidic biosensors with artificial intelligence algorithms to improve the accuracy and reliability of test results and support clinical decision-making.

**Table 4:** An overview of the application of microfluidic biosensors in POCT.

Elements	Content Description
Technical advantages	Low consumption: reduce the amount of reagents used. High sensitivity: improve the sensitivity and accuracy of the test. Portability: easy to carry out in different settings.
Application areas	Inflammation. Infectious diseases. Chronic diseases.
Key challenges	Accuracy and consistency of results. Simplicity and maneuverability of equipment. Application in resource-constrained environments.
Future directions	Optimizing microfluidic chip design to streamline operational steps, enhance device simplicity and operability, and reduce the entry barrier for use. Developing POCT devices capable of detecting multiple biomarkers simultaneously to advance multiparameter testing for a more thorough disease assessment. Integrating POCT equipment with portable devices like smartphones for wireless data transmission and analysis, enabling patient self-monitoring and health management. Exploring new application domains, such as environmental monitoring, food safety, and drug discovery, leveraging the miniaturization, integration, and automation of POCT devices.

## Data Availability

No new data were created or analyzed in this study.
